# Differences in the Tumor Microenvironment between African-American and European-American Breast Cancer Patients

**DOI:** 10.1371/journal.pone.0004531

**Published:** 2009-02-19

**Authors:** Damali N. Martin, Brenda J. Boersma, Ming Yi, Mark Reimers, Tiffany M. Howe, Harry G. Yfantis, Yien Che Tsai, Erica H. Williams, Dong H. Lee, Robert M. Stephens, Allan M. Weissman, Stefan Ambs

**Affiliations:** 1 Laboratory of Human Carcinogenesis, Center of Cancer Research (CCR), National Cancer Institute (NCI), National Institutes of Health (NIH), Bethesda, Maryland, United States of America; 2 Cancer Prevention Fellowship Program, Office of Preventive Oncology, National Cancer Institute (NCI), National Institutes of Health (NIH), Bethesda, Maryland, United States of America; 3 Advanced Biomedical Computing Center, NCI-Frederick/SAIC-Frederick Inc, National Institutes of Health (NIH), Frederick, Maryland, United States of America; 4 Department of Biostatistics, Virginia Commonwealth University, Richmond, Virginia, United States of America; 5 Pathology and Laboratory Medicine, Baltimore Veterans Affairs Medical Center, Baltimore, Maryland, United States of America; 6 Laboratory of Protein Dynamics and Signaling, Center of Cancer Research (CCR), National Cancer Institute (NCI), National Institutes of Health (NIH), Frederick, Maryland, United States of America; University of Cape Town, South Africa

## Abstract

**Background:**

African-American breast cancer patients experience higher mortality rates than European-American patients despite having a lower incidence of the disease. We tested the hypothesis that intrinsic differences in the tumor biology may contribute to this cancer health disparity.

**Methods and Results:**

Using laser capture microdissection, we examined genome-wide mRNA expression specific to tumor epithelium and tumor stroma in 18 African-American and 17 European-American patients. Numerous genes were differentially expressed between these two patient groups and a two-gene signature in the tumor epithelium distinguished between them. To identify the biological processes in tumors that are different by race/ethnicity, Gene Ontology and disease association analyses were performed. Several biological processes were identified which may contribute to enhanced disease aggressiveness in African-American patients, including angiogenesis and chemotaxis. African-American tumors also contained a prominent interferon signature. The role of angiogenesis in the tumor biology of African-Americans was further investigated by examining the extent of vascularization and macrophage infiltration in an expanded set of 248 breast tumors. Immunohistochemistry revealed that microvessel density and macrophage infiltration is higher in tumors of African-Americans than in tumors of European-Americans. Lastly, using an *in silico* approach, we explored the potential of tailored treatment options for African-American patients based on their gene expression profile. This exploratory approach generated lists of therapeutics that may have specific antagonistic activity against tumors of African-American patients, e.g., sirolimus, resveratrol, and chlorpromazine in estrogen receptor-negative tumors.

**Conclusions:**

The gene expression profiles of breast tumors indicate that differences in tumor biology may exist between African-American and European-American patients beyond the knowledge of current markers. Notably, pathways related to tumor angiogenesis and chemotaxis could be functionally different in these two patient groups.

## Introduction

The age-adjusted breast cancer incidence and mortality rates vary substantially among race/ethnic groups [1]. Most notably, European-American women have the highest risk of developing the disease, while African-American women experience the highest mortality rates. This difference in survival between African-American and European-American breast cancer patients has been attributed to differences in socioeconomic factors and access to healthcare. However, after accounting for those differences, African-American women were still found to have lower breast cancer survival rates than European-American women [2]–[5]. The data suggest that having equal medical care may not eliminate the survival disparity between African-American and European-American breast cancer patients, and that other causes are involved in this problem.

It has been proposed that differences in tumor biology may contribute to the survival health disparity associated with breast cancer [6], [7]. Race/ethnic differences in the expression of cell cycle-regulatory proteins in breast tumors have been described [8]. African-American patients also have a greater prevalence of more aggressive, poorly differentiated, estrogen-receptor (ER)-negative tumors and a higher rate of lymph node involvement than European-Americans [4], [5], and they develop breast cancer at an age younger than 35 twice as frequently as European-American women [9]. Recently, a high prevalence of basal-like breast cancers was observed among pre-menopausal African-American breast cancer patients [10], [11]. Because the basal-like subtype is a poor prognosis marker, its increased frequency among African-American patients, when compared with non-African-American patients, could contribute to their disproportionately high breast cancer mortality. However, even after removal of all basal-like cases from the analysis, African-American breast cancer cases still had poorer outcomes than non-African-American cases [10].

We hypothesized that differences exist in the microenvironment of breast tumors comparing African-American with European-American patients. Our laboratory recently observed such differences in prostate cancer and also noted an increased expression of interferon-responsive genes in tumors of African-American men [12]. Analogous to the prostate study, we analyzed the gene expression profiles of breast tumors and used bioinformatics tools to identify differences in oncogenic pathways between the African-American and European-American patients. Guided by the gene expression profiling results, we examined microvessel density and macrophage infiltration in breast tumors by immunohistochemistry. The importance of both for breast cancer growth and spread has been demonstrated [13]–[15]. Using these approaches, we found differences in angiogenesis, chemotaxis, and immunobiology of breast tumors among the two patient groups. In addition, many interferon-regulated genes were found be up-regulated in tumors of African-American patients.

## Results

### Characteristics of study population

Total RNA was isolated from LCM-dissected tumor epithelia and tumor stroma of 35 breast tumors (18 African-American and 17 European-American breast cancer patients). For one African-American patient, LCM did not provide sufficient amounts of good quality RNA from the tumor stroma. Further analyses (e.g., qRT-PCR, Western blotting, and immunohistochemistry) were performed on an extended breast tumor set from 248 breast cancer patients that included 32 of the 35 tumors in the gene expression profiling study. The demographic and clinicopathological data of the 248 patients are shown in Table 1. Clinical characteristics of the LCM-dissected tumors are summarized in Table 2. Consistent with other studies, we observed that African-American women had a higher prevalence of ER-negative and high grade tumors than European-American women.

**Table 1:** Clinical characteristics of the study population.

	All Cases	African-American	European-American	*P* value
	(n = 248)	(n = 143)	(n = 105)	t-test
Age at diagnosis (mean±SD; n = 248)	55.0±13.9	54.4±14.3	55.9±13.2	0.38
	N (%)*	N (%)*	N (%)*	Fisher's exact test
Stage at diagnosis (TNM)				
Stage I	66 (29)	35 (27)	31 (32)	0.56
Stage II	118 (52)	68 (52)	50 (52)	
≥Stage III	44 (19)	28 (21)	16 (16)	
Node status				
Negative	144 (63)	81 (61)	63 (64)	0.68
Positive	86 (37)	51 (39)	35 (36)	
Histology				
Ductal	189 (76)	116 (81)	73 (70)	0.02
Lobular	34 (14)	12 (8)	22 (21)	
Others	25 (10)	15 (11)	10 (9)	
Tumor grade				
Low	34 (16)	17 (13)	17 (20)	0.01
Medium	74 (34)	37 (29)	37 (43)	
High	107 (50)	75 (58)	32 (37)	
Estrogen receptor				
Negative	102 (41)	68 (48)	34 (33)	0.03
Positive	145 (59)	75 (52)	70 (67)	
HER2				
Negative	154 (62)	92 (65)	62 (59)	0.43
Positive	93 (38)	50 (35)	43 (41)	
Nuclear p53				
Negative	173 (70)	92 (64)	81 (77)	0.04
Positive	75 (30)	51 (36)	24 (23)	
Cyclin E				
Negative	181 (74)	97 (70)	84 (81)	0.06
Positive	62 (26)	42 (30)	20 (19)	
Basal-like subtype				
No	191 (82)	106 (79)	85 (87)	0.16
Yes	41 (18)	28 (21)	13 (13)	

* Cases with missing information are not included.

**Table 2:** Clinical characteristics of the microdissected breast tumors.

Characteristic	All cases	African-American	European-American	*P* value^1^
	(n = 35)	(n = 18)	(n = 17)	
Age at diagnosis				
mean±SD^2^ (years)	59±16	56±17	61±15	0.42
Stage at diagnosis (TNM)				
I	4	2	2	
II	29	15	14	1.0
IIIA	2	1	1	
Histology				
Ductal	31	17	14	0.34
Lobular	4	1	3	
Estrogen receptor				
Positive	16	5	11	
Negative	18	13	5	0.04
Unknown	1	0	1	
HER2				
Positive	23	12	11	
Negative	9	4	5	1.0
Unknown	3	2	1	
Nuclear p53				
Positive	10	6	4	
Negative	24	10	14	0.46
Unknown	1	1	0	
Cyclin E				
Positive	15	9	6	
Negative	17	7	10	0.29
Unknown	3	2	1	

1 African-Americans versus European-Americans; t-test or Fisher's exact test (unknown excluded).

2 SD, standard deviation.

### Differences in the gene expression profiles of breast tumors from African-American and European-American women

In an initial analysis, the gene expression profile of the tumor epithelium was compared between African-American and European-American patients. Numerous genes were found to be differentially expressed between them using a FDR-controlled *P*≤0.01 as the cutoff for inclusion into the gene list (Table S1). Consistent with the immunohistochemistry in other studies [8], several cell cycle regulators (e.g., *CDKN2A* (p16), *CCNA2*, *CCNB1*, *CCNE2*) were expressed at significantly higher levels in the tumor epithelium of African-Americans than European-Americans.

**Figure 1:**
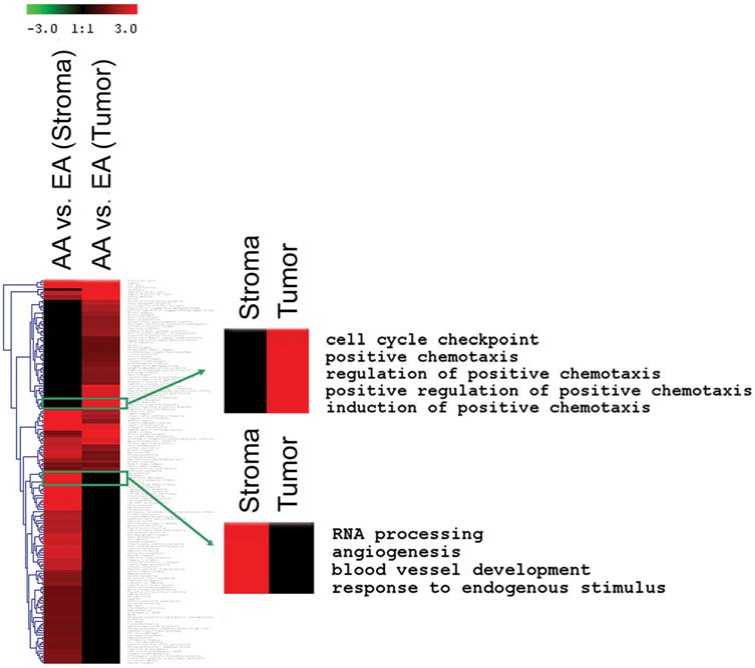
GOBP terms that are enriched for differentially expressed genes in the tumor stroma and the tumor epithelium comparing African-American breast cancer patients (AA, n = 18) with European-American patients (EA, n = 17). The two smaller heatmaps show enlargements of GOBP term clusters that are most significantly enriched for differentially expressed genes (*P*≤0.01) between the two patient groups. Red color intensity is a surrogate for the relative enrichment of differentially expressed genes in a GOBP term. The color coding of the heat maps is related to the enrichment of genes in a biological process (−Log(*P* value)-based) with red indicating a higher enrichment.

**Figure 2:**
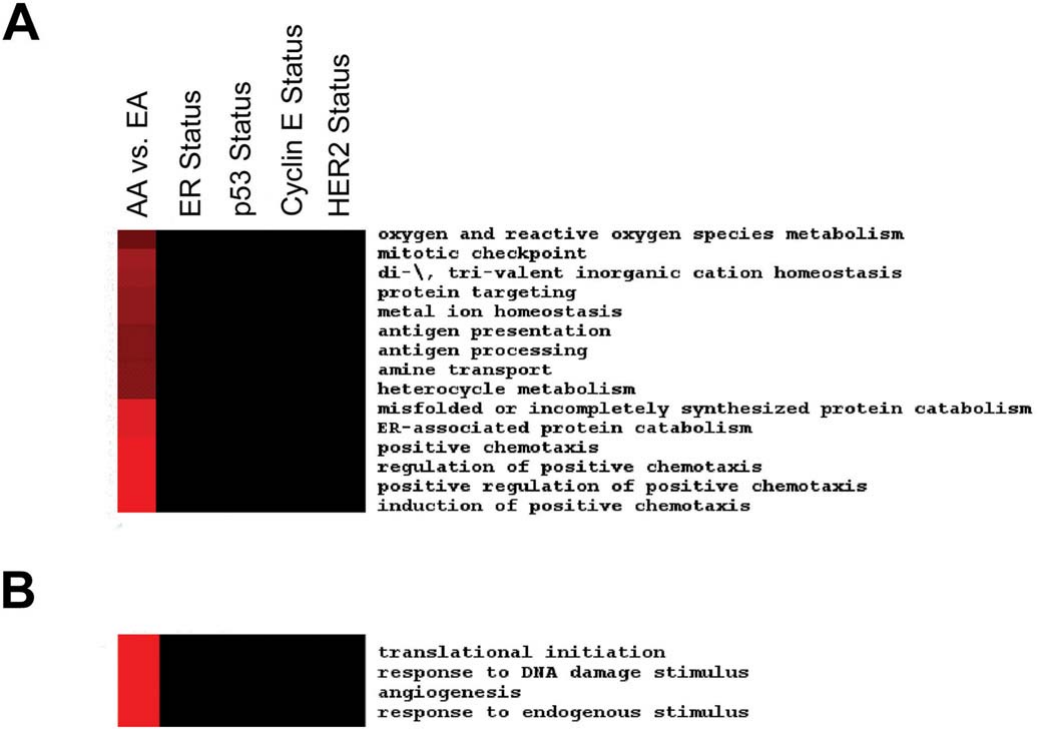
Uniquely enriched biological processes in the tumor epithelium (A) and tumor stroma (B) comparing African-American breast cancer patients with European-American patients. The heatmap shows that some GOBP terms, e.g., those related to endoplasmic reticulum-associated protein catabolism and positive regulation of chemotaxis in the tumor epithelium and angiogenesis in the tumor stroma, show a uniquely strong enrichment for differentially expressed genes when comparing the two patient groups. The same significant enrichments were not observed when we stratified these tumors either by ER status (negative versus positive), p53 status (negative versus positive), cyclin E status (negative versus positive), or HER2 status (positive versus negative). The color coding of the heat maps is related to the enrichment of genes in a biological process (−Log(*P* value)-based) with red indicating a higher enrichment.

As the cumulative effect of multiple genes rather than a single gene effect determines cancer phenotypes, analyses were performed that were aimed to identify biological networks in the microdissected tumor epithelium and tumor stroma that may contribute to the survival health disparity in breast cancer. To find biological processes that differ by race/ethnicity, a cluster analysis was applied to display Gene Ontology biological processes with significant enrichment of those genes that are expressed differentially among the two patient groups (Figure 1). Processes related to cell cycle control and chemotaxis in the tumor epithelium and neovascularization in the tumor stroma were most significantly enriched for differently expressed genes comparing the African-American patients with the European-American patients. Further analysis indicated that the enrichment for differentially expressed genes in these processes was not the product of a confounding influence by other tumor markers. Lists of differentially expressed genes by those markers, e.g., comparing ER-negative with ER-positive tumors or p53 aberrant with p53 wild-type tumors or HER2-negative with HER2-positive tumors, did not yield the same significant enrichment in these processes. Instead, a distinct pattern was observed which denotes biological processes including endoplasmic reticulum-associated degradation and chemotaxis in the tumor epithelium and angiogenesis in the tumor stroma that could be functionally different in African-American and European-American breast tumors (Figure 2). We also assessed whether genes that are differentially expressed between breast tumors from African-American and European-American patients have a common association with other diseases or pathological reactions. We found that those associations exist, e.g., with immune-related reactions (skin-prick test, asthma) and inflammation (Figure S1).

Next, we examined the genes that were most significantly differentially expressed between the African-American and European-American patients independent of the tumors' ER-status (Table 3). Examples of those included *PSPHL*, *TMPO*, *CRYBB2*, and *AMFR* in the tumor epithelium and *PSPHL*, *CXCL10* and *CXCL11* in the tumor stroma. The function of *PSPHL* is unknown while *CRYBB2* encodes a protein linked to congenital eye defects [16]. TMPO is thymopoietin and AMFR is the autocrine mobility factor receptor/gp78. CXCL10 and CXCL11 are ligands of the chemokine receptor CXCR3.

**Table 3:** Most differently expressed genes by race/ethnicity independent of the tumor ER status.

Tumor epithelium							
Gene Name	GenBank ID	Affy ID	ER-negative	*P* value	ER-positive	*P* value	Gene Title
			Fold change*		Fold change*		
PSPHL	NM_003832	205048_s_at	5.0	5×10^−6^	7.2	2×10^−7^	phosphoserine phosphatase-like
TMPO	AW272611	203432_at	1.72	0.0006	1.57	0.006	thymopoietin
AK2	NM_013411	212175_s_at	1.65	0.004	1.67	0.005	adenylate kinase 2
NBN	NM_002485	202907_s_at	1.62	0.002	1.66	0.007	nibrin
CRYBB2	NM_000496	206777_s_at	1.56	0.0005	1.79	0.001	crystallin, beta B2
AMFR	NM_001144	202203_s_at	1.51	0.003	1.59	0.04	Autocrine mobility factor receptor (gp78)
EDG2	AW269335	204036_at	0.64	0.007	0.56	0.005	lysophosphatidic acid receptor 2
PLAGL1	NM_002656	207002_s_at	0.5	0.003	0.64	0.004	pleiomorphic adenoma gene-like 1 (Zac1)

Tumor stroma							
Gene Name	GenBank ID	AffyID	ER-negative	*P* value	ER-positive	*P* value	Gene Title
			Fold change*		Fold change*		
PSPHL	NM_003832	205048_s_at	4.59	5×10^−6^	5.96	7×10^−9^	phosphoserine phosphatase-like
CXCL10	NM_001565	204533_s_at	5.65	9.6×10^−5^	2.1	0.01	Chemokine (C-X-C motif) ligand 10
CXCL11	AF002985	211122_s_at	1.96	0.007	2.9	0.0002	Chemokine (C-X-C motif) ligand 11
SLC38A1	AK024263	218237_s_at	1.9	0.001	2.71	0.005	solute carrier family 38, member 1
PAICS	BX538303	201014_s_at	1.73	0.009	1.92	0.003	phosphoribosylaminoimidazole carboxylase
PSMA2	BX641097	201317_s_at	1.62	0.001	1.63	0.005	proteasome subunit, alpha type, 2
AK2	NM_013411	212175_s_at	1.6	0.002	1.5	0.01	adenylate kinase 2
LAPTM4B	BC038117	208767_s_at	1.6	0.01	1.66	0.007	Lysosomal associated protein transmembrane 4 beta
LASS6	NM_203463	212446_s_at	1.6	0.007	1.67	0.009	LAG1 longevity assurance homolog 6
SOS1	AL833457	212777_at	1.57	0.00007	1.82	0.0002	son of sevenless homolog 1
SMS	AA552813	202043_s_at	1.57	0.004	1.87	0.007	spermine synthase
ARF4	BC016325	201096_s_at	1.54	0.01	1.6	0.007	ADP-ribosylation factor 4
C7orf24	BF570959	215380_s_at	1.51	0.005	2.4	0.0009	chromosome 7 open reading frame 24
CDC42BPA	NM_003607	214464_at	0.64	0.009	0.43	0.0006	CDC42 binding protein kinase alpha
TGFB1I1	AK122975	209651_at	0.63	0.002	0.54	0.003	TGF beta1 induced transcript 1
PTRF	NM_012232	208790_s_at	0.62	0.001	0.65	0.003	polymerase I and transcript release factor
NUAK1	AB011109	204589_at	0.62	0.0009	0.54	0.008	NUAK family, SNF1-like kinase, 1
PLAGL1	NM_002656	207002_s_at	0.6	0.003	0.42	0.01	pleiomorphic adenoma gene-like 1 (Zac1)
AQP1	NM_198098	209047_at	0.53	0.007	0.45	0.009	aquaporin 1

* At least 1.5-fold difference in expression and *P*<0.05 comparing African-American versus European-American patients ( = reference).

**Figure 3:**
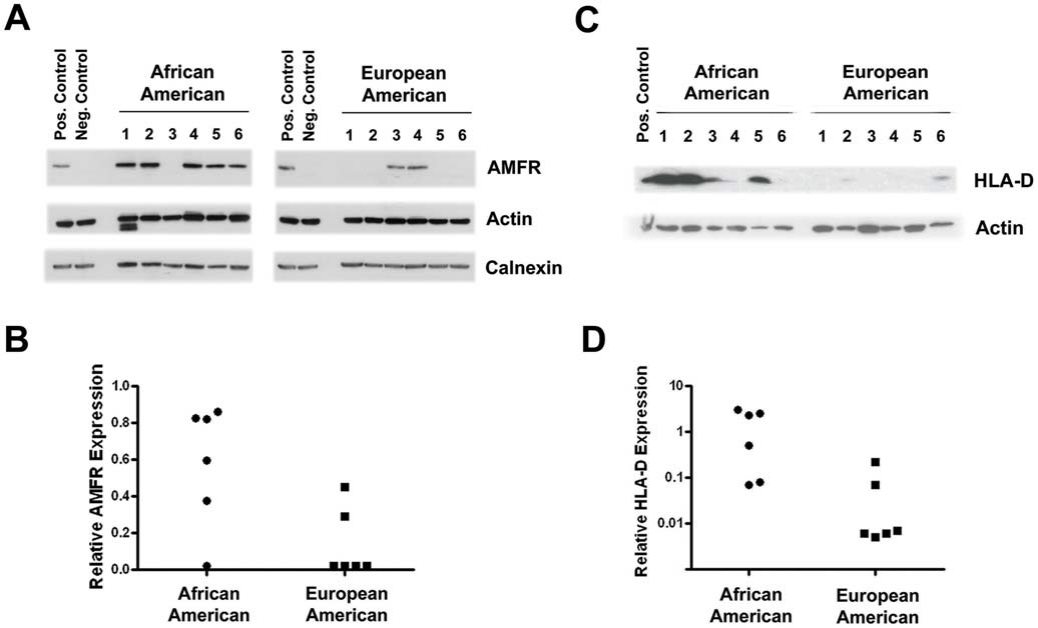
Autocrine mobility factor receptor (AMFR) and HLA-D protein expression in breast tumors. (A) Shown is the protein expression of AMFR in extracts of 6 tumors from African-American patients and 6 tumors from European-American patients. β-actin and calnexin are loading controls. Like AMFR, calnexin is located in the endoplasmic reticulum. (B) Quantification of AMFR expression. Shown is the relative expression of AMFR protein after normalization to β-actin. AMFR expression is significantly higher in tumors from African-American patients than in tumors from European-American patients (*P* = 0.038; Wilcoxon rank sum test). (C) Differential HLA-D protein expression in ER-negative breast tumors. Shown is the expression of HLA-D in extracts of 6 tumors from African-American patients and 6 tumors from European-American patients. (D) Quantification of HLA-D expression. Shown is the relative expression of HLA-D protein after normalization to β-actin. The expression is significantly higher in tumors from African-American patients than in tumors from European-American patients (*P* = 0.016; Wilcoxon rank sum test).

*AMFR* is a candidate oncogene that promotes metastasis [17] and a key gene in endoplasmic reticulum-associated degradation, which was identified as a race/ethnicity-related biological process in this study. To corroborate the *AMFR* microarray data, we followed up by Western blot analysis. AMFR protein expression was determined in tumor extracts from 6 African-American and 6 European-American patients that were randomly selected from available fresh-frozen tumor tissues. As shown in Figure 3, AMFR was significantly higher expressed in African-American tumors than in European-American tumors.

We previously had demonstrated that *PSPHL* and *CRYBB2* could be used as a two gene classifier to differentiate between African-American and European-American prostate cancer patients [12]. To test if similar signatures exist in breast cancer, we applied the prediction analysis of microarray algorithm to interrogate our datasets for genes that can differentiate between African-American and European-American breast cancer patients. In accordance with the prostate study, the two gene signature consisting of probesets for *PSPHL* (205048_s_at) and *CRYBB2* (206777_s_at) was identified as the top-ranked predictor that can distinguish between the breast tumor epithelia from African-Americans and European-Americans (Table 4). Ninety-four percent of the African-American patients and all European-American patients were correctly classified by the expression pattern of the two genes. This result was confirmed in 55 additional breast tumors (27 African-American and 28 European-American breast cancer patients) using qRT-PCR quantification of *PSPHL* and *CRYBB2* expression. In this validation set, the two-gene signature correctly classified 93% of the African-American patients and 86% of the European-American patients.

**Table 4:** Classification of tumors by race/ethnicity with a two-gene signature consisting of *PSPHL* and *CRYBB2*.

	Test set (microarray-based expression)			
True/Predicted	African-American	European-American	Total	% Accuracy
African-American	17	1	18	94
European-American	0	17	17	100

	Validation set (qRT-PCR-based expression)			
True/Predicted	African-American	European-American	Total	% Accuracy
African-American	25	2	27	93
European-American	4	24	28	86

For the tumor stroma, a five gene classifier consisting of probesets for *PSPHL* (205048_s_at), *CXCL10* (204533_at), *CXCL11* (211122_s_at), *ISG20* (204698_at), and *GMDS* (204875_s_at) was identified. The expression pattern of these five stromal genes correctly classified 16 out of 17 African-Americans (94%) and 14 out of 17 European-Americans (82%). Notably, three genes in the classifier for the tumor stroma, *CXCL10*, *CXCL11*, and *ISG20*, are known interferon γ-regulated genes. An interferon gene signature was also present in the profiles derived from the tumor epithelium of the ER-positive tumors (e.g., *STAT1*, *IFIT1*, *IFIH1*, *IFI27*, *ISG15*, *OAS1*, *OAS3*, *OASL*; Table S2) and ER-negative tumors (e.g., *HLA-DQA1* and *HLA-DQB1*; Table S3). The presence of this signature was further supported by a Gene Set Enrichment Analysis (GSEA) that revealed significant associations between the parenchymal signature in the ER-positive tumors and published signatures derived from interferon (α,β,γ)-treated cells (Table 5).

**Table 5:** Highest-ranked GSEA-annotated terms enriched for genes that are differently expressed by race/ethnicity in ER-positive tumors.

GSEA Term	GSEA Hits*	All Genes‡	Annotated Genes in GSEA dataset†	All Annotated Genes††	Fisher's exact test *P* value
1. Up-regulated in fibroblasts at 6 hours following treatment with interferon-alpha	21	243	52	11852	1.6E-22
2. Effect of NUP98-HOXA9 on gene transcription at 3 d after transduction (UP)	30	243	182	11852	4.7E-19
3. Genes up-regulated by interferon-alpha in primary hepatocyte	15	243	52	11852	7.1E-14
4. Up-regulated in fibroblasts at 6 hours following either infection with UV-inactivated CMV or interferon-alpha	12	243	28	11852	9.6E-14
5. Genes up-regulated by interferon-beta in HT1080 (fibrosarcoma)	18	243	94	11852	4.9E-13
6. Up-regulated in fibroblasts following infection with human cytomegalovirus (at least 3-fold, with Affymetrix change call, in at least two consecutive timepoints), with maximum change at 12 hours	11	243	26	11852	1.3E-12
7. Genes up-regulated by interferon-alpha in HT1080 (fibrosarcoma)	15	243	66	11852	3.3E-12
8. Genes significantly up-regulated in SLE patient blood mononuclear cells	11	243	29	11852	5.4E-12
9. Up-regulated at any timepoint up to 24 hours following infection of HEK293 cells with reovirus strain T3Abney	24	243	234	11852	7.6E-11
10. Genes up-regulated by interferon-gamma in colon,derm,iliac,aortic,lung endothelial cells	14	243	71	11852	1.4E-10
11. Upregulated 2-fold in HT1080 cells 6 hours following treatment with any of interferons alpha, beta and gamma	13	243	83	11852	1.2E-08

* Annotated genes in the GSEA dataset that are differently expressed (P≤0.01) comparing ER-positive tumors from African-American and European-American women.

‡ All GSEA-annotated genes that are differently expressed comparing ER-positive tumors from African-American and European-American women.

† All annotated genes in this GSEA dataset.

†† All GSEA-annotated genes.

**Figure 4:**
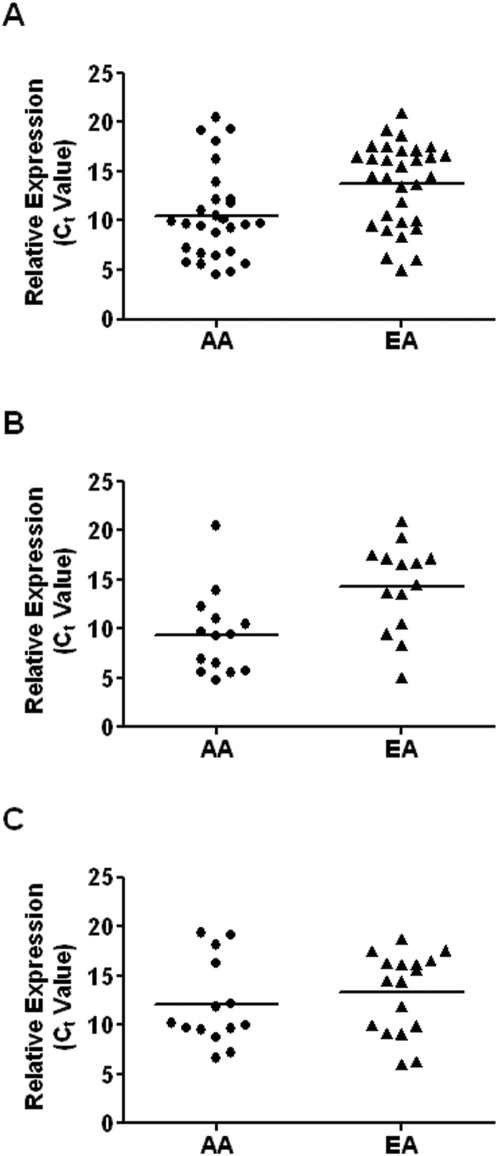
Differences in tumor *HLA-DQ* mRNA expression between African-American (AA) and European-American (EA) breast cancer patients. qRT-PCR analysis of *HLA-DQ* expression in extracts from 59 fresh-frozen breast tumors. (A) Relative *HLA-DQ* expression comparing AA patients (n = 28) with EA patients (n = 31) across all tumors. The expression of *HLA-DQ* is significantly higher in AA than EA patients (*P* = 0.007; t-test). (B) Relative *HLA-DQ* expression in ER-negative tumors comparing AA patients (n = 14) with EA patients (n = 14). The expression of *HLA-DQ* is significantly higher in AA than EA patients (average fold difference: 29.6; *P* = 0.006; t-test). (C) Relative *HLA-DQ* expression in ER-positive tumors comparing AA patients (n = 14) with EA patients (n = 17). The expression of *HLA-D* mRNA in ER-positive tumors was not significantly different between AA and EA patients (*P* = 0.42; t-test). Note: C_t_ values are inversely correlated to mRNA expression.

The most differently expressed genes in ER-negative tumors between African-American and European-American women were HLA-D family members, *HLA-DQA1* and *HLA-DQB1*. The up-regulation of the MHC II antigen (HLA-D) in the ER-negative African-American breast cancer patients was corroborated by Western blot analysis (Figure 3). The microarray data did not show that *HLA-DQ* is significantly differentially expressed by race/ethnicity in the ER-positive tumors. We confirmed this finding by qRT-PCR analysis in a validation set consisting of 59 tumors (Figure 4).

### Microvessel density, tumor-associated macrophages and T-regulatory cells

**Figure 5:**
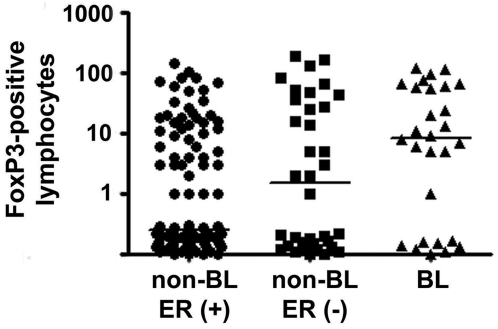
FoxP3-positive lymphocytes in breast tumors. The number of FoxP3-positive lymphocytes is highest in basal-like breast tumors (BL; n = 28) when compared to estrogen receptor (ER)-positive breast tumors (non-BL, ER (+); n = 111) and non-basal-like ER-negative breast tumors (non-BL, ER (−), n = 36). *P* for trend: 0.0004. Shown is the average FoxP3-positive lymphocyte count per 250× field. Solid lines in graph indicate the median. Basal-like breast tumors are defined as being ER-negative, HER2-negative, cytokeratin 5-positive and/or HER1-positive.

Our previous analyses indicated that biological processes related to chemotaxis and tumor angiogenesis could be functionally different between breast tumors from African-American patients and European-American patients. Known inducers of angiogenesis, such as vascular endothelial growth factor and syndecan-1, were among the genes that were higher expressed in the tumor epithelium of African-American patients than European-American patients (Table S1). Thus, we examined microvessel density, a surrogate for angiogenesis, and the number of tumor-associated macrophages (TAM) and FoxP3-positive T-regulatory cells in breast tumors by immunohistochemistry and assessed their relation to the race/ethnic background of the patients. Both TAM and T-regulatory cells have previously been associated with increased angiogenesis in breast tumors [14], [18]. African-American patients (n = 125) were significantly more likely than European-American patients (n = 83) to have a high tumor vessel density (*P* = 0.047; Fisher's exact test). This relationship was partly confounded by differences in age at diagnosis, tumor ER status, TNM stage, and grade of the tumors among the two patient groups, as indicated by a logistic regression analysis (Table 6). African-American patients (n = 142) were also significantly more likely than European-American patients (n = 105) to have a high TAM count (*P* = 0.01; Fisher's exact test). This relationship was independent of the age at diagnosis, tumor ER status, and disease stage and grade (Table 6). A significant correlation between microvessel density and TAMs existed in tumors of African-American women (rho = 0.45; *P*<0.0001; Spearman rank correlation) and tumors of European-American women (rho = 0.22; *P* = 0.04; Spearman rank correlation). After adjusting for age at diagnosis, tumor ER status, and disease stage and grade, the association remained significant only in African-American patients. No association was observed between the number of FoxP3-postive T-regulatory cells in breast tumors and race/ethnicity. However, our analysis revealed a significant association between an increased number of these cells and the basal-like breast cancer subtype (Figure 5).

**Table 6:** Association of microvessel density and macrophage count with race/ethnicity of patients.

	Logistic regression (unadjusted) OR (95% CI)	Logistic regression (adjusted**) OR (95% CI)
High microvessel density*		
European-American	1 (reference)	1
African-American	1.83 (1.05 to 3.22)	1.70 (0.91 to 3.22)
High macrophage count*		
European-American	1	1
African-American	1.99 (1.19 to 3.33)	1.91 (1.05 to 3.49)

* CD31-positive microvessels and CD68-positive macrophages were dichotomized into low/high at the median.

** Odds ratio (OR) adjusted by age at diagnosis, tumor ER status, tumor grade, TNM stage.

### Connectivity Map to identify putative antagonistic small molecule therapeutics

**Figure 6:**
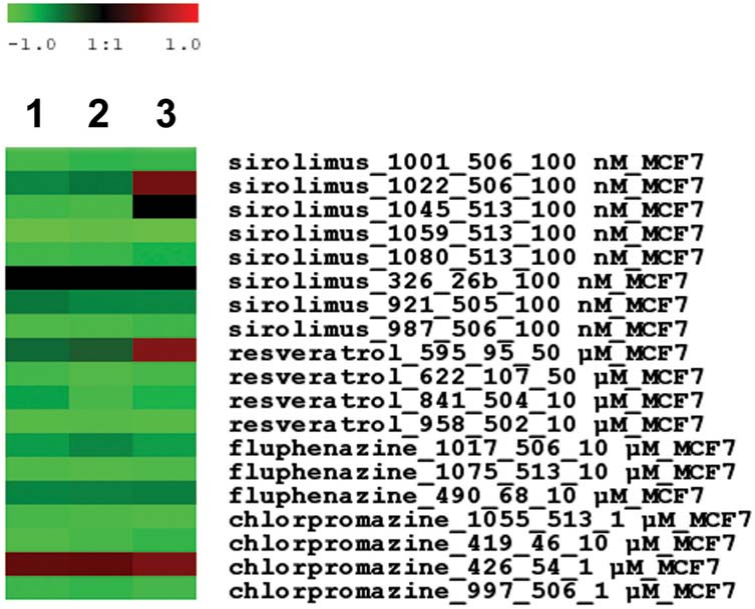
Connections between gene signatures induced by small molecule drugs in MCF-7 breast cancer cells and a query signature. The query signature consisted of the differentially expressed genes in ER-negative breast tumors comparing African-American with European-American patients. Shown is a heatmap with the color-coded connectivity score (−1 to +1) for individual sets of experiments exposing MCF-7 cells to sirolimus, resveratrol, or two phenothiazines, fluphenazine and chlorpromazine. The heatmap shows that negative correlations between drug-induced signatures and the query signature were found for multiple experiments with MCF-7 cells. Green: negative connectivity indicating an antagonist effect of the drug on the query signature. Black: no connectivity. Red: positive connectivity indicating an agonist effect. Different cutoffs were used for inclusion of differentially expressed genes in the query signature: (1) *P*≤0.01, (2) *P*≤0.01 and fold change at least 1.5-fold, (3) *P*≤0.05 and fold change at least 1.5-fold. The negative correlations between signatures induced by sirolimus, resveratrol, or chlorpromazine, and the query signature were statistically significant at *P_permutation_*<0.05, respectively, in the combined analysis with n = 8 for sirolimus, n = 4 for resveratrol, and n = 4 for chlorpromazine.

We explored the potential to tailor treatment options for African-American patients based on their gene expression profiles. The Connectivity Map database is a collection of genome-wide transcriptional expression data from cell lines, including MCF-7 breast cancer cells, which were treated with a panel of bioactive small molecules [19]. Integrated pattern-matching algorithms enable the discovery of functional connections between these molecule-induced signatures and disease-induced signatures through the feature of common gene expression changes. A significant negative correlation between a molecule-induced signature and disease-induced signature indicates that this molecule could potentially reverse the disease signature. We used this database to identify small molecules that may reverse the gene expression signature in the tumor epithelium of breast tumors that differentiates African-American women from European-American women. By this mechanism, these compounds may also target the survival health disparity. The analysis revealed a significant negative correlation between gene signatures in MCF-7 cells induced by LY-294002 (*P_permutation_* = 0.0002), a PI3-kinase inhibitor, and Y-27632 (*P_permutation_* = 0.008), a Rho signaling inhibitor, and the genes that are differentially expressed between African-American and European-American women in ER-positive tumors. The analysis for ER-negative tumors revealed significant negative correlations between the signatures induced by sirolimus (rapamycin) (*P_permutation_* = 0.006), an immunosuppressant drug, resveratrol (*P_permutation_* = 0.046), an antibacterial and antifungal phytoalexin, and chlorpromazine (*P_permutation_* = 0.025), a phenothiazine, and the genes that are differentially expressed between African-American and European-American women in these tumors (Figure 6). The results indicate that these compounds may potentially antagonize the gene expression profiles in ER-positive and ER-negative tumors that differentiate African-American breast cancer patients from European-American breast cancer patients. We did not apply this same analysis to the tumor stroma signature because the MCF7 therapy response could be very different from the therapy response of stromal cells.

## Discussion

We used gene expression profiling to identify biological differences in the microdissected tumor epithelium and tumor stroma that may exist between African-American and European-American breast cancer patients from the greater Baltimore area. African-Americans from this area, and for most of the U.S., have ancestral links to specific regions in West Africa and experience an increased frequency of aggressive breast cancer similar to West African women [20].

Our study focused on the discovery of differentially regulated biological processes comparing African-American and European-American breast cancer patients as opposed to the discovery of specific genes. This approach was used as it may reveal differences in oncogenic pathways and the tumor microenvironment, and therefore may provide insight into therapeutic opportunities [12], [21]. Using this design, we found that biological processes related to chemotaxis, angiogenesis, endoplasmic reticulum function, and cell cycle control were most significantly enriched for genes that are differentially expressed by race/ethnicity. Of those processes, only the cell cycle control association appeared to be significantly confounded by differences in other tumor markers such as the race/ethnic difference in the tumor ER status.

One of our most interesting observations was the presence of an interferon signature in tumors of the African-American patients. The rationale of examining our datasets for an interferon signature was based on our previous observation in prostate cancer showing that interferon γ-responsive genes are up-regulated in African-American tumors [12]. Interferons are commonly induced by pathogens and is a key pro-inflammatory cytokine in inflammation and autoimmune disease. In tumor biology, interferon γ is the master regulator of the Th1 response and enhances tumor immunogenicity and abrogated tumor development in mouse models [22]. However, an interferon γ signature can be merely an indicator of a chronic inflammation and has been observed as a component of breast cancer progression in HER2-transgenic mice [23]. Currently, we are uncertain why breast tumors from African-American patients have an interferon signature, or why interferon-regulated genes were more differentially expressed in the ER-positive than ER-negative tumors, although this may reflect estrogenic regulation of host immunity [24]. The signature in the tumor epithelium could partly be caused by infiltrating immune cells, which cannot be completely separated from the tumor epithelial cells by LCM. Several of the interferon-related genes in our tumor signature have previously been reported as key genes in the cellular defense against bacterial and viral pathogens [25]–[27] and in the promotion of all steps of breast tumorigenesis including tumor development, growth, survival, and metastasis [28]–[30]. We hypothesize that etiologic agents may induce the signature in breast and prostate tumors of African-Americans. Alternatively, chronic inflammation and/or specific genetic variations in immune-related genes could be more prevalent among these African-American cancer patients and cause the heightened interferon activity in their tumors.

There have been two recent reports that investigated gene expression variations between individuals with European ancestry and individuals with African ancestry (Nigeria) using lymphoblastoid cell lines [31], [32]. Analogous to the present study, the authors assessed the enrichment of biological processes and pathways by genes that are differentially expressed by race/ethnicity. Notably, processes related to antimicrobial humoral response, inflammation mediated by chemokines and cytokines, histamine H1 receptor-mediated signaling pathway, toll-receptor signaling pathway, and the VEGF signaling pathway were identified. The results from these two studies suggest that differences in the genetic background between healthy volunteers of European ancestry and those from Nigeria cause gene expression differences affecting host immune response, inflammation and chemotaxis, and angiogenesis. These findings are consistent with our findings in breast tumors, and while preliminary, raise the possibility that differences in common genetic variations among African-American and European-American breast cancer patients may lead to group-specific alterations in cancer-related pathways that control host response, inflammation, and tumor angiogenesis.

Few others have studied race/ethnic differences in the expression of tumor markers in breast cancer and observed that p16, p53, and cyclin E were more commonly expressed in tumors from African-American patients than European-American patients [8], [33]. p16 is encoded by *CDKN2A*. This gene was also up-regulated in the African-American tumors of this study, as indicated by the microarray data. Nuclear accumulation of p53 protein in tumor cells is a surrogate for a functional impairment of the p53 pathway while the overexpression of cyclin E is most frequently caused by a post-transcriptional mechanism that leads to the accumulation of hyperactive low molecular weight cyclin E isoforms [34], [35]. Consistent with the previous reports, p53 and cyclin E protein accumulation was also more common in African-American tumors than European-American tumors in the present study.

The gene expression profiles of breast tumors indicated that pathways related to tumor angiogenesis and chemotaxis could be functionally different between African-American and European-American patients. For further corroboration of these findings, we demonstrated higher levels of microvessel density and TAMs in African-American tumors than European-American tumors. Increased microvessel density and the infiltration of tumors by macrophages have been shown to be poor prognosis markers [13]–[15]. The two markers are interrelated as TAMs are a major source of chemokines and cytokines which induce tumor angiogenesis [14], [15]. From the present study, we do not know why TAM infiltration is increased in tumors from African-American women. Possibly, tumors from African-American patients release more chemotactic cytokines to attract the infiltration of TAM than those from European-American patients. Among the most important of the chemotactic signals that attract TAM are MCP-1, CSF-1, and VEGF [15]. Of those, VEGF was found to be higher expressed in the African-American tumors. We also assessed the infiltration of tumors by FoxP3-positive T-regulatory cells. The number of these cells in breast tumors has been associated with an increased risk of relapse in breast cancer [36]. Although the number of these immune suppressive cells was not significantly different between African-American and European-American breast tumors, elevated numbers of these cells were found in basal-like tumors. Future studies should investigate whether these T-regulatory cells are related to poor outcome among young African-American breast cancer patients who frequently present with the basal-like subtype [10], [11].

If gene expression differences exist between African-American and European-American breast cancer patients, those differences could potentially be exploited for tailored therapy. In an exploratory approach, we used the Connectivity Map database to identify small molecules that may antagonize the gene expression signature in the tumor epithelium of breast tumors that differentiated African-American patients from European-American patients. An antagonist could potentially improve the therapeutic outcome of high risk breast cancer patients, e.g., African-American patients with ER-negative tumors. Significant negative connectivity scores, indicating an antagonistic effect of the small molecule drug on the query signature, were obtained for the PI3-kinase inhibitor, LY-294002, and Rho signaling inhibitor, Y-27632, in ER-positive breast tumors and for sirolimus, resveratrol, and chlorpromazine in ER-negative tumors. Although preliminary, these findings are novel and may have implications for cancer therapy. The toxicity profile of LY-294002 excludes it from clinical trials, but its antagonist effect suggests that ER-positive African-American and European-American breast cancer patients could respond differentially to therapeutic PI3-kinase inhibitors. Sirolimus is rapamycin, which is a mTOR inhibitor and an immunosuppressant. Sirolimus and other mTOR inhibitors are currently in clinical trials, and their anti-tumor efficacy in breast cancer in preclinical models has been demonstrated [37], [38]. Resveratrol is a chemopreventive agent that has a plethora of effects in breast cancer including anti-inflammatory activities, apoptosis induction, angiogenesis inhibition, and effects on PI3-kinase signaling, among others [39], [40]. Chlorpromazine is a psychotropic agent and not an anti-cancer drug. However, recent data have shown that chlorpromazine has anti-proliferative and pro-apoptotic effects in cancer cells [41], [42], and future studies should evaluate the activity of chlorpromazine in breast cancer.

Our study has strengths and limitations. We conducted a microarray analysis of microdissected tumor epithelium and tumor stroma, thus providing comprehensive databases of gene expression for these two cellular compartments in African-American and European-American breast tumors. The use of LCM also largely ruled out that varying amounts of tumor and stroma between African-American and European-American tumors is a confounder of the datasets. Despite the advantages of more rigorous histological sampling enabled by LCM, the approach has limitations. LCM is labor intensive and cannot be applied for the analyses of large sample sets, and further stratification of the breast tumors in the many subtypes (basal-like, ERBB+, normal-like, luminal A & B & C) was not possible in this sample set because of sample size limitations.

In conclusion, the gene expression profiles of breast tumors correspond to differences in tumor biology between African-American and European-American patients. Particularly, pathways related to chemotaxis, tumor angiogenesis, and immunobiology could be functionally different in the two patient groups. The therapeutic implications of those differences should be evaluated in future studies.

## Materials and Methods

### Tissue collection

Fresh-frozen (n = 35) and paraffin-embedded (n = 248) tumor specimens were obtained from breast cancer patients that resided in the greater Baltimore area, as described previously [43], [44]. Patients were recruited at the University of Maryland Medical Center (UMD), the Baltimore Veterans Affairs Medical Center, Union Memorial Hospital, Mercy Medical Center, and the Sinai Hospital in Baltimore between 1993 and 2003. All patients were identified through surgery lists and enrolled into the study prior to surgery. They signed a consent form and completed an interviewer-administered questionnaire. These patients had pathologically confirmed breast cancer, were of African-American or European-American descent by self-report, were diagnosed with breast cancer within the last 6 months before recruitment, and had, by self-report, no previous history of the disease. Clinical and pathological information, including tumor ER status, was obtained from medical records and pathology reports. Disease staging was performed according to the tumor–node–metastasis (TNM) system of the American Joint Committee on Cancer/the Union Internationale Contre le Cancer (AJCC/UICC). The Nottingham system was used to determine the tumor grade. The collection of tumor specimens, survey data, and clinical and pathological information was reviewed and approved by the University of Maryland Institutional Review Board for the participating institutions (UMD protocol #0298229). IRB approval of this protocol was then obtained at all institutions (Veterans Affairs Medical Center, Union Memorial Hospital, Mercy Medical Center, and Sinai Hospital). The research was also reviewed and approved by the NIH Office of Human Subjects Research (OHSR #2248).

### Laser capture microdissection

Enriched tumor epithelium and tumor stroma from 35 fresh-frozen surgical breast tumors was obtained by laser capture microdissection (LCM) as described [44]. In brief, frozen eight-micron serial sections from OCT-preserved frozen tissues were prepared and mounted on plain, uncharged microscope slides. One Hematoxylin/eosin-stained section of each specimen was reviewed by a pathologist before commencing dissection. The pathologist indicated which representative sections of the tumors should be microdissected. LCM was performed at the NIH Collaborative Research LCM Core Laboratory with the Pixcell II LCM system (Arcturus, Mountain View, CA). At least 3000 to 5000 cells were obtained per specimen. Total RNA was isolated using the PicoPure protocol (Arcturus, Mountain View, CA). The mRNA was amplified with two linear amplification steps by *in vitro* transcription using the MEGAscript T7 kit (Ambion, Austin, TX) followed by the labeling step using the BioArray HighYield RNA Transcript Labeling Kit T3 from Enzo Life Sciences (Farmingdale, NY). Labeled cRNA was hybridized onto Affymetrix HG-U133A GeneChips. Cel files with the normalized expression data, and additional tumor marker information, were deposited in the GEO repository (GSE5847). Table S4 lists the GEO accession number for the Cel files of each sample.

### Analysis of gene expression data

All chips were normalized with the robust multichip analysis procedure [45]. Gene lists comparing expression of tumor epithelium from African-Americans to tumor epithelium from European-Americans and tumor stroma from African-Americans to tumor stroma from European-Americans were generated using moderated t-scores to obtain *P* values that are false discovery rate (FDR)-controlled [44]. Prediction analysis for microarrays (PAM) was used to classify patients as either African-American or European-American [46]. Pathway and disease association analyses were performed with the in-house WPS software [47] and a *P*≤0.01 cutoff point for inclusion of differentially expressed genes. Biological processes were annotated according to Gene Ontology Biological Processes (GOBP) (Gene Ontology Consortium: http://www.geneontology.org). A one-sided Fisher's exact test was used to determine which biological processes had a statistically significant enrichment of differentially expressed genes. If the microarray analysis yielded several significantly differentially expressed transcripts that all encoded one gene, only one entry of this gene was used for significance testing. We then compiled the Fisher's exact test results for cluster analyses and displayed the results in color-coded heat maps to reveal the patterns of significantly altered biological processes and pathways. The color coding of our heatmaps is related to the enrichment of genes in a biological process/pathway (−Log(*P* value)-based) with red indicating a higher enrichment. A disease association analysis was conducted using both our WPS software and information provided by the genetic association database (http://geneticassociationdb.nih.gov). In this analysis, we assessed whether differentially expressed genes between breast tumors from African-American and European-American patients had previously been associated with other diseases, as indicated by the genetic association database, and whether these disease-associated genes, were significantly enriched in the breast tumor gene signature using the one-sided Fisher's exact test. The results were displayed in a heatmap. We also performed a Gene Set Enrichment Analysis [48] to find significant overlaps between race/ethnicity-related gene signatures in breast cancer and published signatures archived in the molecular signature database (http://www.broad.mit.edu/gsea/msigdb/).

### Connectivity mapping

Functional connections between gene signatures derived from the comparison between African-American and European-American breast patients and gene signatures induced by small molecules (164 molecules that target cancer and other diseases) were explored using the Connectivity Map database at http://www.broad.mit.edu/cmap. The database is a collection of genome-wide transcriptional expression data from cultured cells treated with bioactive small molecules at different concentrations and pattern-matching algorithms that enable the discovery of functional connections between drugs, genes and diseases [19]. The significance of a negative or positive correlation (permutated *P* value) between a query signature, e.g., a list of differentially expressed genes (*P*≤0.01) from the comparison between African-American and European-American breast cancer patients, and the expression data from a cell line treated with a small molecule is calculated based on the likelihood that the correlation between these two lists is a product of chance as described in the manual of cmap.

### Quantitative Real-Time PCR

Total RNA was extracted from 59 fresh-frozen breast tumors (bulk tissue) using the Trizol method and subjected to reverse transcription for quantitative PCR (qRT-PCR). Twenty-eight of the tumors were from African-American patients and 31 were from European-American patients. None of those tumors was used in the gene expression profiling study. qRT-PCR was subsequently performed in duplicate using the TaqMan *PSPHL*, *CRYBB2*, and *HLA-DQ* expression assays (Applied Biosystems, Foster City, CA), which include pre-optimized probes and primer sets for these genes. The *CRYBB2* assay was designed to specifically target the Affymetrix microarray probeset for this gene. Data were collected using the ABI PRISM® 7500 Sequence Detection System. The 18 s RNA was used as the internal standard reference. Normalized expression was calculated using the comparative C_t_ method and fold changes were derived from the 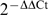 values for each gene. A power analysis was performed with the publicly available PS software [49], [50] to assess that the chosen sample size was sufficient to detect a 1.5-fold difference or greater in expression between the two patient groups with an assumed standard deviation of 0.6 and α<0.05 in a two-sided t-test. With these assumptions, we had a power >90% to detect a statistically significant difference between 28 African-American tumors and 31 European-American tumors.

### Protein extraction and Western blot analyses

Fresh-frozen tumor samples were crushed into small pieces in liquid nitrogen and homogenized in 2 ml of M-PER lysis buffer containing protease inhibitors and iodoacetamide (all Pierce Biotechnology, Rockford, IL). Protein concentrations were determined with the Bio-Rad Protein Assay (Bio-Rad Laboratories, Hercules, CA). Western blot analysis was performed according to standard procedures and 50 µg of total protein were loaded per lane. The following antibodies were used to visualize the membrane-bound proteins: 1∶100 diluted mouse monoclonal antibody for HLA-D (MHC class II antigen) (Abcam, Cambridge, MA); 1 µg/ml rabbit polyclonal antibody (Ab2) for autocrine mobility factor receptor (gp78) [17].

### Immunohistochemistry

Formalin-fixed and paraffin-embedded 5 µm slides were deparaffinized and placed into citrate buffer for antigen retrieval. To detect FoxP3, slides were pre-treated with trypsin for 10 min at 37°C prior to antigen retrieval. Slides were microwaved and rinsed in 1× phosphate-buffered saline (PBS) buffer. Endogenous peroxidase was blocked using the DakoCytomation Envision System-HRP blocking buffer according to the manufacturer's protocol (DakoCytomation, Carpinteria, CA). After an overnight incubation with the primary antibody at 4°C, slides were washed in PBS and incubated with a corresponding HRP-labeled secondary antibody using the DakoCytomation Envision System reagents. Slides were washed after 30 min incubation at room temperature, were stained with DAB, and counterstained with Methyl Green. Marker expression was evaluated using the following primary antibodies: 1∶100 diluted monoclonal DO-7 antibody (DakoCytomation) for p53; 1∶100 diluted rabbit polyclonal antibody (DakoCytomation) for c-erbB-2 (HER2); ready-to-use monoclonal Ab-2 (Clone HE12, cross-reacts with the C-terminus) antibody (Lab Vision Corp., Fremont, CA) for cyclin E; ready-to-use mouse monoclonal Ab-1 (Clone JC/70A) antibody for CD31 (Lab Vision Corp., Fremont, CA); ready-to-use mouse monoclonal Ab-3 antibody for CD68 (Lab Vision Corp.); ready-to-use mouse monoclonal antibody for CD138/syndecan-1 (Lab Vision Corp.); 1∶1500 diluted rabbit polyclonal antibody for FoxP3 (Abcam); 1∶250 diluted mouse monoclonal antibody for cytokeratin 5 (Lab Vision Corp.); and ready-to-use mouse monoclonal antibody for EGFR (HER1) (Lab Vision Corp.). Nuclear p53 expression was scored positive if more than 10% of the tumor cells expressed nuclear p53, as described [44], [51]. To score the immunohistochemistry of cyclin E (nuclear), cytokeratin-5 (cytosolic), EGFR (membrane-bound), and HER2 (membrane-bound), a combined score of intensity and distribution was used to categorize the immunohistochemical staining for protein expression according to a standard protocol [52], [53]. Intensity received a score of 0 to 3 if the staining was negative, weak, moderate, or strong. The distribution received a score of 0 to 4 if the staining distribution was <10% positive cells, 10%–30%, >30%–50%, >50%–80%, and >80%. A sum score was then divided into four groups as follows: (1) negative = 0–1, (2) weak = 2–3, (3) moderate = 4–5, and (4) strong = 6–7. Negative to weak staining was scored as being negative. Moderate to strong nuclear staining was scored as being positive.

### Basal-like breast cancer

In accordance with the literature [10], basal-like breast tumors were defined as being ER-negative and HER2-negative, cytokeratin 5-positive and/or HER1-positive. The ER status was obtained from medical records. Basal-like breast cancers, as defined by these criteria, were significantly associated with an aberrant tumor p53 status and accumulation of nuclear cyclin E expression (each *P*<0.001). Both have previously been described as markers of basal-like breast cancers [54], [55].

### Microvessel and cell counts

The quantification of the tumor microvessel density was performed on CD31-positive microvessels according to the method of *Weidner et al.* [13]. If the available tumor section did not contain representative fields for the count, the tumor was not scored. The number of macrophages and T regulatory cells in the tumor specimens was determined by counting the number of CD68-positive macrophages and FoxP3-positive T-regulatory cells per 250× field in 3 representative fields. If the available tumor section did not contain 3 representative fields for the count, the tumor was not scored.

### Statistical analysis

Stata 7.0 (Stata Corp, College Station, TX) statistical software was used for data analysis. Statistical tests were two-sided and an association was considered statistically significant with *P*<0.05. The t-test and the Wilcoxon rank sum test were applied to analyze the relationship between race/ethnicity and continuous data. The Spearman correlation coefficient was calculated for correlation analyses of continuous and categorical data. The *P* for trend was determined by Spearman rank correlation. The χ^2^ and Fisher's exact tests were used to analyze dichotomized data and multivariate logistic regression was used to calculate odds ratios. To generate dichotomized data for immunohistochemistry, scores were divided into moderate to strong versus negative to weak, or into strong versus negative-moderate. Continuous data, such as CD31 and CD68 counts, were dichotomized into low and high using the median value as the cutoff.

## Supporting Information

Figure S1 Disease association analysis. Shown is a heat map. The list of differentially expressed genes (P≤0.01) comparing tumor epithelium from African-American breast cancer patients (AA) with tumor epithelium from European-American patients (EA) was analyzed for their relationship with other diseases using the genetic association database. The red color indicates common associations between differentially expressed genes in a gene list, e.g., AA versus EA (all tumors), and other diseases. The disease association analysis was performed for four gene lists: AA (n = 18) versus EA (n = 17) for all tumors combined; AA (n = 13) versus EA (n = 5) for ER-negative tumors, AA (n = 5) versus EA (n = 11) for ER-positive tumors and ER-positive (n = 16) versus ER-negative tumors (n = 18). Red color intensity is a surrogate for the strength of the association.
(0.09 MB PDF)

Table S1 (0.04 MB PDF)

Table S2 (0.03 MB PDF)

Table S3 (0.02 MB PDF)

Table S4 (0.01 MB PDF)
