# Supplementary figures and images for: Differences in the Tumor Microenvironment between African-American and European-American Breast Cancer Patients
Source: PLoS One. 2009 Feb 19;4(2):e4531. doi: 10.1371/journal.pone.0004531 (PMC2638012; doi:10.1371/journal.pone.0004531)

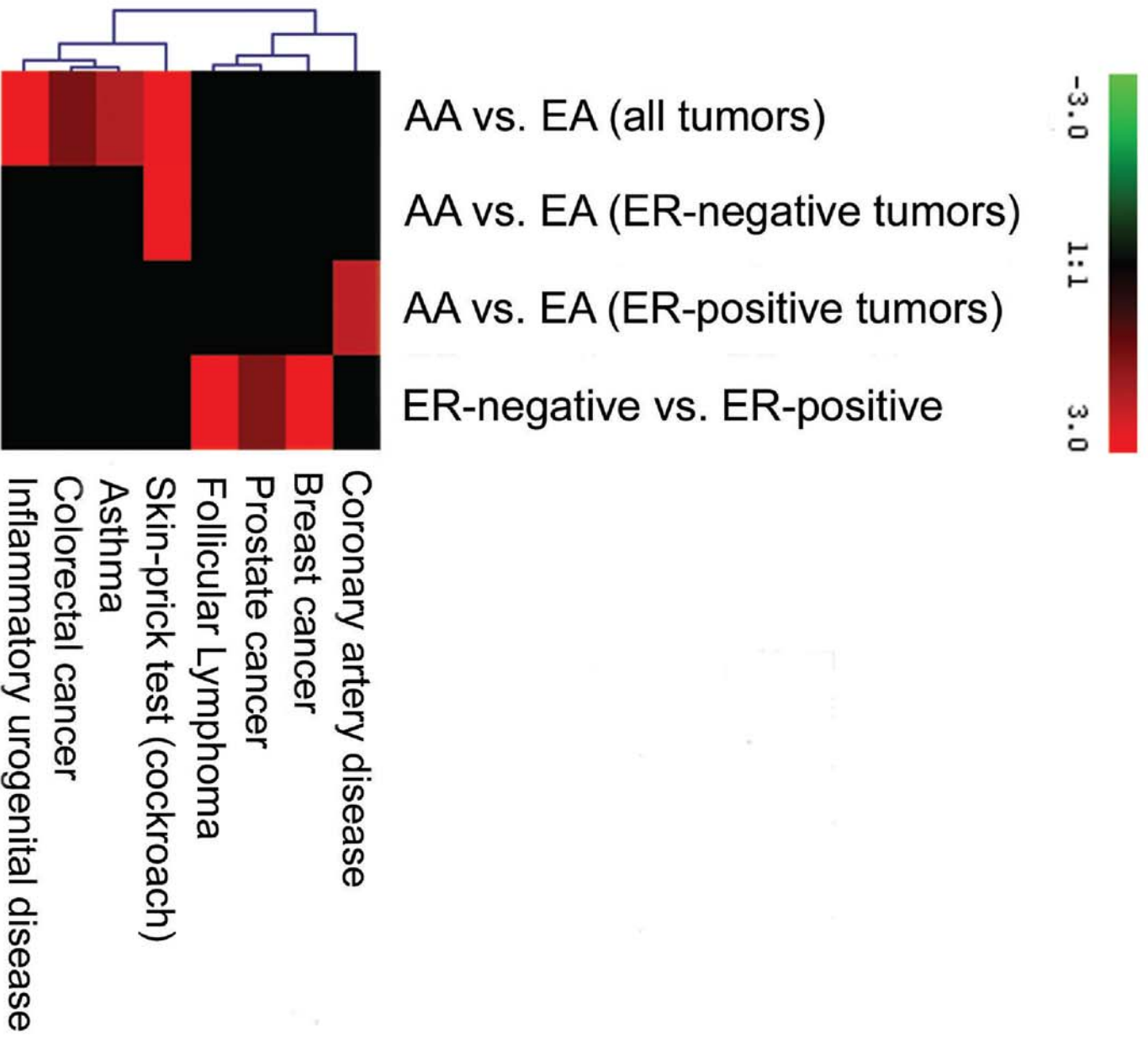

Supplement: Figure S1 — Disease association analysis. Shown is a heat map. The list of differentially expressed genes (P≤0.01) comparing tumor epithelium from African-American breast cancer patients (AA) with tumor epithelium from European-American patients (EA) was analyzed for their relationship with other diseases using the genetic association database. The red color indicates common associations between differentially expressed genes in a gene list, e.g., AA versus EA (all tumors), and other diseases. The disease association analysis was performed for four gene lists: AA (n = 18) versus EA (n = 17) for all tumors combined; AA (n = 13) versus EA (n = 5) for ER-negative tumors, AA (n = 5) versus EA (n = 11) for ER-positive tumors and ER-positive (n = 16) versus ER-negative tumors (n = 18). Red color intensity is a surrogate for the strength of the association. (0.09 MB PDF) [file pone.0004531.s001.pdf]
