# Supplementary material for: Differences in the Tumor Microenvironment between African-American and European-American Breast Cancer Patients
Source: PLoS One. 2009 Feb 19;4(2):e4531. doi: 10.1371/journal.pone.0004531 (PMC2638012; doi:10.1371/journal.pone.0004531)
Supplement: Table S1 — (0.04 MB PDF) [file pone.0004531.s002.pdf]

Table S1. Differently expressed genes in the LCM-dissected tumor epithelium comparing African-American and European-American breast cancer patients

Gene Symbol	GenBank ID	Affy ID	Fold change	*P* value	Gene Title
ABCA8	NM_007168	204719_at	0.54	5.81E-03	ATP-binding cassette, sub-family A (ABC1), member 8
ABCF1	NM_001090	200045_at	1.30	2.48E-03	ATP-binding cassette, sub-family F (GCN20), member 1
ABHD2	AI557319	63825_at	0.65	2.05E-03	abhydrolase domain containing 2
	BE671816	221815_at	0.67	3.27E-03	abhydrolase domain containing 2
ABLIM3	NM_014945	205730_s_at	0.72	3.59E-03	actin binding LIM protein family, member 3
ACACB	AI970898	43427_at	0.64	6.66E-03	acetyl-Coenzyme A carboxylase beta
	AI057637	221928_at	0.71	8.46E-03	acetyl-Coenzyme A carboxylase beta
ACP5	NM_001611	204638_at	0.54	3.74E-03	acid phosphatase 5, tartrate resistant
ACTR3B	NM_020445	218868_at	1.47	2.80E-03	ARP3 actin-related protein 3 homolog B (yeast)
ADCY9	AB011092	204497_at	0.64	6.08E-03	adenylate cyclase 9
ADH1A	AF153821	209614_at	0.82	8.78E-03	alcohol dehydrogenase 1A (class I), alpha polypeptide
ADH1B	M24317	209612_s_at	0.49	1.35E-03	alcohol dehydrogenase IB (class I), beta polypeptide
	M21692	209613_s_at	0.57	7.83E-03	alcohol dehydrogenase IB (class I), beta polypeptide
AGTR1	NM_000685	205357_s_at	0.32	4.97E-05	angiotensin II receptor, type 1
AK2	AL513611	212175_s_at	1.42	4.36E-03	adenylate kinase 2
ALDH3A2	NM_000382	202054_s_at	0.66	2.18E-03	aldehyde dehydrogenase 3 family, member A2
AMFR	NM_001144	202203_s_at	1.55	1.00E-03	autocrine motility factor receptor
AOF2	AB011173	212348_s_at	1.34	4.00E-03	amine oxidase (flavin containing) domain 2
AOX1	NM_001159	205083_at	0.69	1.08E-03	aldehyde oxidase 1
APBA2	AB014719	209871_s_at	1.35	8.58E-04	amyloid beta (A4) precursor protein-binding, family A, member 2 (X11-like)
APOL1	AF323540	209546_s_at	1.45	3.94E-03	apolipoprotein L, 1
APOL6	NM_030641	219716_at	1.50	6.30E-04	apolipoprotein L, 6
APP	X06989	214953_s_at	1.34	6.45E-03	amyloid beta (A4) precursor protein (peptidase nexin-II, Alzheimer disease)
ARF4	AL537042	201096_s_at	1.42	8.41E-03	ADP-ribosylation factor 4
ASAH1	AI934569	213702_x_at	0.71	1.90E-03	N-acylsphingosine amidohydrolase (acid ceramidase) 1
ATP2C1	AF225981	209935_at	1.43	3.69E-03	ATPase, Ca++ transporting, type 2C, member 1
	AF189723	211137_s_at	1.55	3.97E-03	ATPase, Ca++ transporting, type 2C, member 1
ATP6AP2	NM_005765	201444_s_at	0.73	6.44E-03	ATPase, H+ transporting, lysosomal accessory protein 2
AURKA	NM_003158	208079_s_at	1.69	4.25E-03	aurora kinase A

	NM_003600	204092_s_at	1.44	6.27E-03	aurora kinase A
B4GALT5	BF691447	221484_at	1.56	4.23E-04	UDP-Gal:betaGlcNAc beta 1,4-galactosyltransferase, polypeptide 5
BANF1	AF044773	210125_s_at	1.38	8.18E-03	barrier to autointegration factor 1
BIRC1	AI817801	204860_s_at	0.71	9.34E-03	baculoviral IAP repeat-containing 1
BIRC5	NM_001168	202095_s_at	1.63	3.03E-03	baculoviral IAP repeat-containing 5 (survivin)
BNIP3L	AF060922	221479_s_at	0.71	1.20E-03	BCL2/adenovirus E1B 19kDa interacting protein 3-like
	AL132665	221478_at	0.71	8.77E-03	BCL2/adenovirus E1B 19kDa interacting protein 3-like
BPGM	NM_001724	203502_at	1.58	1.14E-03	2,3-bisphosphoglycerate mutase
BTN3A2	BC002832	209846_s_at	1.52	9.34E-03	butyrophilin, subfamily 3, member A2
BUB1	AF043294	209642_at	1.44	6.35E-03	BUB1 budding uninhibited by benzimidazoles 1 homolog (yeast)
BUB1B	NM_001211	203755_at	1.58	2.38E-03	BUB1 budding uninhibited by benzimidazoles 1 homolog beta (yeast)
C11orf41	H08993	214772_at	1.31	6.46E-03	chromosome 11 open reading frame 41
C14orf132	NM_020215	218820_at	0.58	7.05E-04	chromosome 14 open reading frame 132
C1GALT1	NM_020156	219439_at	1.33	2.02E-03	core 1 synthase, glycoprotein-N-acetylgalactosamine 3-beta-galactosyltransferase, 1
C1orf103	NM_018372	220235_s_at	1.40	2.99E-03	chromosome 1 open reading frame 103
C1orf63	AF247168	209006_s_at	0.73	3.82E-03	chromosome 1 open reading frame 63
C2orf17	AK026155	222129_at	0.68	2.02E-04	Chromosome 2 open reading frame 17
C7orf24	AK021779	215380_s_at	1.57	2.46E-03	chromosome 7 open reading frame 24
C9orf76	NM_024945	218979_at	1.40	2.19E-03	chromosome 9 open reading frame 76
CAPN6	NM_014289	202965_s_at	1.53	9.31E-03	calpain 6
CARM1	AA551784	212512_s_at	1.26	5.94E-03	coactivator-associated arginine methyltransferase 1
CBS	BE613178	212816_s_at	1.27	8.05E-03	cystathionine-beta-synthase
CCDC47	NM_020198	217814_at	1.40	9.49E-03	coiled-coil domain containing 47
CCDC59	NM_014167	218936_s_at	1.29	5.26E-03	coiled-coil domain containing 59
CCL5	M21121	1405_i_at	1.74	2.32E-03	chemokine (C-C motif) ligand 5
CCNA2	AI346350	213226_at	1.43	5.85E-03	Cyclin A2
CCNB1	BE407516	214710_s_at	1.97	1.17E-03	cyclin B1
CCNE2	NM_004702	205034_at	1.55	4.48E-03	cyclin E2
CCNI	AF135162	208656_s_at	0.79	7.35E-03	cyclin I
CCT6A	NM_001762	201327_s_at	1.49	2.43E-03	chaperonin containing TCP1, subunit 6A (zeta 1)
CD302	NM_014880	203799_at	0.54	1.53E-04	CD302 molecule
CD47	Z25521	211075_s_at	1.54	3.98E-03	CD47 molecule
CDC20	NM_001255	202870_s_at	1.83	7.77E-03	CDC20 cell division cycle 20 homolog (S. cerevisiae)
CDC25A	AI343459	204695_at	1.33	7.17E-03	cell division cycle 25A
CDC37L1	NM_017913	219343_at	1.40	5.32E-03	CDC37 cell division cycle 37 homolog (S. cerevisiae)-like 1
CDC42BPA	NM_003607	214464_at	0.56	9.89E-04	CDC42 binding protein kinase alpha (DMPK-like)
	NM_014826	203794_at	0.76	8.45E-03	CDC42 binding protein kinase alpha (DMPK-like)
CDH1	NM_004360	201131_s_at	2.51	3.76E-03	cadherin 1, type 1, E-cadherin (epithelial)
CDH3	NM_001793	203256_at	1.97	3.36E-03	cadherin 3, type 1, P-cadherin (placental)
CDKN2A	NM_000077	207039_at	2.31	9.77E-05	cyclin-dependent kinase inhibitor 2A (melanoma, p16, inhibits CDK4)

	U38945	209644_x_at	1.87	1.26E-04	cyclin-dependent kinase inhibitor 2A (melanoma, p16, inhibits CDK4)
CENPA	NM_001809	204962_s_at	2.08	1.73E-04	centromere protein A, 17kDa
CEP55	NM_018131	218542_at	1.91	2.01E-03	centrosomal protein 55kDa
CEP57	NM_014679	203494_s_at	0.77	5.10E-03	centrosomal protein 57kDa
CFD	NM_001928	205382_s_at	0.39	1.88E-04	complement factor D (adipsin)
CGI-38	NM_016140	218876_at	0.73	5.18E-03	brain specific protein
CHD7	NM_017780	218829_s_at	1.42	2.45E-03	chromodomain helicase DNA binding protein 7
CHEK1	NM_001274	205394_at	1.44	2.75E-03	CHK1 checkpoint homolog (S. pombe)
CHRD	AF283325	211248_s_at	0.74	5.72E-03	chordin
CKS2	NM_001827	204170_s_at	1.84	1.00E-03	CDC28 protein kinase regulatory subunit 2
CLK1	AI251890	214683_s_at	0.73	5.79E-03	CDC-like kinase 1
COL13A1	M33653	211343_s_at	1.47	7.20E-03	collagen, type XIII, alpha 1
COL9A3	NM_001853	204724_s_at	1.54	5.27E-03	collagen, type IX, alpha 3
COLEC12	NM_030781	221019_s_at	0.53	8.12E-04	collectin sub-family member 12
CRIP1	NM_001311	205081_at	0.50	5.77E-03	cysteine-rich protein 1 (intestinal)
CRYBB2	NM_000496	206777_s_at	1.80	1.44E-07	crystallin, beta B2
CRYL1	NM_015974	220753_s_at	0.77	5.61E-03	crystallin, lambda 1
CTA-246H3.1	AL022324	215946_x_at	2.28	1.53E-04	similar to omega protein
CXCL10	NM_001565	204533_at	2.94	2.39E-04	chemokine (C-X-C motif) ligand 10
CXCL11	AF030514	210163_at	3.17	1.02E-04	chemokine (C-X-C motif) ligand 11
	AF002985	211122_s_at	2.38	5.65E-04	chemokine (C-X-C motif) ligand 11
CYBRD1	NM_024843	217889_s_at	0.56	3.34E-04	cytochrome b reductase 1
CYCS	BC005299	208905_at	1.32	1.94E-03	cytochrome c, somatic
CYP2J2	NM_000775	205073_at	1.35	5.70E-03	cytochrome P450, family 2, subfamily J, polypeptide 2
DACH1	NM_004392	205472_s_at	0.65	8.36E-03	dachshund homolog 1 (Drosophila)
DBF4	NM_006716	204244_s_at	1.58	4.07E-05	DBF4 homolog (S. cerevisiae)
DDEF2	NM_003887	206414_s_at	1.50	4.58E-03	development and differentiation enhancing factor 2
DDIT4	NM_019058	202887_s_at	1.76	4.08E-03	DNA-damage-inducible transcript 4
DDX17	NM_030881	208151_x_at	1.54	4.90E-03	DEAD (Asp-Glu-Ala-Asp) box polypeptide 17
	AW188131	213998_s_at	0.62	8.82E-03	DEAD (Asp-Glu-Ala-Asp) box polypeptide 17
DDX58	NM_014314	218943_s_at	1.89	2.51E-04	DEAD (Asp-Glu-Ala-Asp) box polypeptide 58
DHRS1	AL050217	213279_at	0.79	7.30E-03	dehydrogenase/reductase (SDR family) member 1
DLG7	NM_014750	203764_at	1.66	4.34E-03	discs, large homolog 7 (Drosophila)
DNAJC15	NM_013238	218435_at	1.62	4.11E-03	DnaJ (Hsp40) homolog, subfamily C, member 15
DNM1L	NM_012062	203105_s_at	1.39	6.75E-03	dynamin 1-like
DPP8	NM_017743	220939_s_at	0.77	1.45E-03	dipeptidyl-peptidase 8
DSC2	NM_004949	204751_x_at	1.65	4.97E-03	desmocollin 2
DSG2	BF031829	217901_at	1.85	6.87E-04	desmoglein 2
DSP	NM_004415	200606_at	2.68	3.27E-03	desmoplakin
DUSP14	NM_007026	203367_at	0.81	8.99E-03	dual specificity phosphatase 14
DUT	U62891	208956_x_at	0.74	2.55E-03	dUTP pyrophosphatase
E2F5	U15642	221586_s_at	1.37	3.06E-03	E2F transcription factor 5, p130-binding
ECGF1	NM_001953	204858_s_at	1.50	2.41E-03	endothelial cell growth factor 1 (platelet-derived)

ECM1	U65932	209365_s_at	0.61	9.33E-04	extracellular matrix protein 1
ECT2	NM_018098	219787_s_at	1.71	2.95E-03	epithelial cell transforming sequence 2 oncogene
EDG2	AW269335	204036_at	0.67	2.10E-03	endothelial differentiation, lysophosphatidic acid G-protein-coupled receptor, 2
	BF055366	204037_at	0.65	2.75E-03	endothelial differentiation, lysophosphatidic acid G-protein-coupled receptor, 2
EFEMP1	AI826799	201842_s_at	0.49	5.71E-03	EGF-containing fibulin-like extracellular matrix protein 1
EFHC1	NM_018100	219833_s_at	0.52	1.91E-05	EF-hand domain (C-terminal) containing 1
EHF	NM_012153	219850_s_at	1.52	7.80E-03	ets homologous factor
EIF3S9	U78525	208688_x_at	1.31	6.88E-04	eukaryotic translation initiation factor 3, subunit 9 eta, 116kDa
	NM_003751	203462_x_at	1.31	1.26E-03	eukaryotic translation initiation factor 3, subunit 9 eta, 116kDa
	BC001173	211501_s_at	1.31	4.25E-03	eukaryotic translation initiation factor 3, subunit 9 eta, 116kDa
EIF5	BG481972	208705_s_at	0.78	6.69E-03	eukaryotic translation initiation factor 5
EIF5B	AB018284	201025_at	1.29	2.09E-03	eukaryotic translation initiation factor 5B
	BE138647	214313_s_at	1.29	4.31E-03	eukaryotic translation initiation factor 5B
ENOSF1	AF305057	213645_at	0.66	2.02E-03	enolase superfamily member 1
EPHA4	NM_004438	206114_at	0.71	1.17E-03	EPH receptor A4
ESPL1	NM_012291	204817_at	1.35	2.45E-03	extra spindle poles like 1
	D79987	38158_at	1.33	5.43E-03	extra spindle poles like 1
ETFA	NM_000126	201931_at	1.62	3.95E-03	electron-transfer-flavoprotein, alpha polypeptide (glutaric aciduria II)
ETV5	NM_004454	203349_s_at	1.32	6.35E-03	ets variant gene 5 (ets-related molecule)
EVA1	AF275945	203780_at	1.52	3.49E-03	epithelial V-like antigen 1
EZH2	NM_004456	203358_s_at	1.76	6.00E-04	enhancer of zeste homolog 2
F8	NM_000132	205756_s_at	0.73	2.18E-03	coagulation factor VIII, procoagulant component (hemophilia A)
FABP4	NM_001442	203980_at	0.31	5.08E-04	fatty acid binding protein 4, adipocyte
FABP7	NM_001446	205030_at	4.28	6.26E-05	fatty acid binding protein 7, brain
	NM_001446	205029_s_at	2.71	8.96E-05	fatty acid binding protein 7, brain
FAM46A	AW246673	221766_s_at	0.63	5.91E-03	family with sequence similarity 46, member A
FAM60A	NM_021238	220147_s_at	1.67	1.27E-03	family with sequence similarity 60, member A
FAM98A	AL049943	212333_at	1.56	3.40E-04	family with sequence similarity 98, member A
FBL	M30448	211623_s_at	1.44	9.66E-03	fibrillarin
FBXO5	NM_012177	218875_s_at	1.41	6.32E-04	F-box protein 5
FGF13	NM_004114	205110_s_at	1.85	1.88E-03	fibroblast growth factor 13
FHL1	NM_001449	201540_at	0.55	8.68E-03	four and a half LIM domains 1
FLJ12505	NM_024749	219740_at	1.45	7.73E-03	hypothetical protein FLJ12505
FLJ21062	NM_024788	219455_at	0.80	8.32E-03	hypothetical protein FLJ21062
FMOD	NM_002023	202709_at	0.66	1.27E-03	fibromodulin
FNBP1L	AW270932	215017_s_at	1.41	5.94E-03	formin binding protein 1-like
FOLH1	AW168915	215363_x_at	1.40	6.33E-03	folate hydrolase (prostate-specific membrane antigen) 1
	NM_004476	205860_x_at	1.43	8.36E-03	folate hydrolase (prostate-specific membrane antigen) 1
FOS	BC004490	209189_at	0.43	8.44E-04	v-fos FBJ murine osteosarcoma viral oncogene homolog
FOXK2	NM_004514	203064_s_at	1.41	6.48E-03	forkhead box K2

FOXM1	NM_021953	202580_x_at	1.77	9.33E-04	forkhead box M1
FRAT2	AB045118	209864_at	1.42	2.09E-03	frequently rearranged in advanced T-cell lymphomas 2
FRMD4B	AU145019	213056_at	0.71	3.95E-03	FERM domain containing 4B
FTSJ3	NM_017647	218103_at	1.29	6.74E-03	FtsJ homolog 3 (E. coli)
GABBR1	NM_006398	205890_s_at	2.39	1.83E-03	gamma-aminobutyric acid (GABA) B receptor, 1; ubiquitin D
GABBR2	AF056085	209990_s_at	1.55	2.34E-03	gamma-aminobutyric acid (GABA) B receptor, 2
GADD45G	NM_006705	204121_at	0.70	4.55E-03	growth arrest and DNA-damage-inducible, gamma
GALNT3	BF063271	203397_s_at	2.58	6.94E-05	UDP-N-acetyl-alpha-D-galactosamine:polypeptide N-acetylgalactosaminyltransferase 3 (GalNAc-T3)
GAPVD1	AK023841	212802_s_at	0.81	5.43E-03	GTPase activating protein and VPS9 domains 1
GARS	D30658	208693_s_at	1.37	1.56E-03	glycyl-tRNA synthetase
GCH1	NM_000161	204224_s_at	1.57	4.72E-03	GTP cyclohydrolase 1 (dopa-responsive dystonia)
GGH	NM_003878	203560_at	2.13	4.95E-04	gamma-glutamyl hydrolase (conjugase, folylpolygammaglutamyl hydrolase)
GINS1	NM_021067	206102_at	1.64	2.78E-03	GINS complex subunit 1 (Psf1 homolog)
GLI3	NM_000168	205201_at	0.76	1.85E-03	GLI-Kruppel family member GLI3 (Greig cephalopolysyndactyly syndrome)
GLT8D1	NM_018446	218147_s_at	0.63	8.86E-04	glycosyltransferase 8 domain containing 1
	NM_018446	218146_at	0.78	5.56E-03	glycosyltransferase 8 domain containing 1
GLUL	AL161952	215001_s_at	0.75	7.52E-04	glutamate-ammonia ligase (glutamine synthetase)
GMDS	AI762113	214106_s_at	1.66	1.79E-05	GDP-mannose 4,6-dehydratase
	NM_001500	204875_s_at	2.02	5.69E-05	GDP-mannose 4,6-dehydratase
GMNN	NM_015895	218350_s_at	1.54	5.92E-03	geminin, DNA replication inhibitor
GNB5	NM_016194	204000_at	0.72	4.60E-04	guanine nucleotide binding protein (G protein), beta 5
GNE	NM_005476	205042_at	1.47	8.70E-03	glucosamine (UDP-N-acetyl)-2-epimerase/N-acetylmannosamine kinase
GPIAP1	NM_005898	200723_s_at	1.31	2.02E-03	GPI-anchored membrane protein 1
GPX4	NM_002085	201106_at	0.73	3.95E-03	glutathione peroxidase 4 (phospholipid hydroperoxidase)
GSDML	NM_018530	219233_s_at	1.86	5.04E-04	gasdermin-like
GSTT1	NM_000853	203815_at	0.61	5.57E-03	glutathione S-transferase theta 1
HCFC1R1	AA436930	45714_at	0.78	3.58E-03	host cell factor C1 regulator 1 (XPO1 dependent)
HIP1R	AB013384	209558_s_at	1.27	4.73E-03	huntingtin interacting protein 1 related
HIST1H2BG	BC001131	210387_at	1.77	7.46E-03	histone 1, H2bg
HLA-DQA1	X00452	213831_at	2.92	7.98E-04	major histocompatibility complex, class II, DQ alpha 1
HLA-DQB1	M16276	209480_at	2.19	4.67E-03	major histocompatibility complex, class II, DQ beta 1
HMGA1	NM_002131	206074_s_at	1.35	6.79E-03	high mobility group AT-hook 1
HMGB3	NM_005342	203744_at	1.91	1.43E-03	high-mobility group box 3
HN1	NM_016185	217755_at	1.71	6.40E-03	hematological and neurological expressed 1
HNMT	BC005907	211732_x_at	0.72	3.60E-03	histamine N-methyltransferase

HNRPA3	BG505670	211931_s_at	1.35	8.68E-03	heterogeneous nuclear ribonucleoprotein A3
	AA528233	211933_s_at	1.34	8.82E-03	heterogeneous nuclear ribonucleoprotein A3
HRASLS	NM_020386	219983_at	1.82	5.25E-03	HRAS-like suppressor
HSD17B4	NM_000414	201413_at	0.68	5.81E-03	hydroxysteroid (17-beta) dehydrogenase 4
HYI	NM_031207	221435_x_at	0.77	2.65E-03	hydroxypyruvate isomerase homolog
ICAM3	NM_002162	204949_at	1.38	6.65E-03	intercellular adhesion molecule 3
IDS	NM_006123	206342_x_at	0.76	4.89E-03	iduronate 2-sulfatase (Hunter syndrome)
IDUA	NM_000203	205059_s_at	0.71	8.40E-04	iduronidase, alpha-L-
IFIH1	NM_022168	219209_at	1.90	1.56E-03	interferon induced with helicase C domain 1
IGH	M87789	211430_s_at	4.07	4.57E-04	immunoglobulin heavy locus
	BG340548	214916_x_at	1.54	1.57E-03	immunoglobulin heavy locus
IGHA1	S55735	217022_s_at	7.37	1.61E-05	immunoglobulin heavy alpha 1
IGHD	AI858004	213674_x_at	1.68	4.39E-04	immunoglobulin heavy constant delta
IGHM	BC001872	209374_s_at	4.17	4.21E-05	immunoglobulin heavy constant mu
	U80139	216491_x_at	1.78	1.28E-03	immunoglobulin heavy constant mu
IGJ	AV733266	212592_at	3.65	2.82E-05	Immunoglobulin J polypeptide
IGKC	BG485135	214669_x_at	3.32	3.15E-05	Immunoglobulin kappa constant
	BC005332	221651_x_at	3.38	4.61E-05	immunoglobulin kappa constant
	M63438	221671_x_at	3.40	4.87E-05	immunoglobulin kappa constant
	BG536224	214836_x_at	2.75	1.37E-04	immunoglobulin kappa constant
	L14458	211644_x_at	2.84	3.18E-04	immunoglobulin kappa constant
IGKV1D-13	AW408194	216207_x_at	2.30	6.18E-04	immunoglobulin kappa variable 1D-13
IGL	AV698647	215379_x_at	4.04	1.50E-05	immunoglobulin lambda locus
	X57812	214677_x_at	5.78	2.32E-05	immunoglobulin lambda locus
	AA680302	215121_x_at	4.20	2.43E-05	immunoglobulin lambda locus
	M87790	209138_x_at	5.16	2.47E-05	Immunoglobulin lambda locus
	AF234255	216853_x_at	1.48	2.12E-03	Immunoglobulin lambda locus
IGLC2	AF047245	216365_x_at	1.36	2.20E-03	immunoglobulin lambda constant 2
IGLJ3	AB001733	211798_x_at	1.80	3.49E-03	immunoglobulin lambda joining 3
	AB014341	211881_x_at	1.68	5.91E-03	immunoglobulin lambda joining 3
IGLV2-14	AJ249377	217148_x_at	1.80	8.93E-03	immunoglobulin lambda variable 2-14
IL13RA1	NM_001560	201887_at	0.73	4.24E-03	interleukin 13 receptor, alpha 1
IL1R1	NM_000877	202948_at	0.58	5.77E-03	interleukin 1 receptor, type I
IL6R	NM_000565	205945_at	0.63	2.88E-03	interleukin 6 receptor
ILF3	AF141870	211375_s_at	1.39	1.03E-03	interleukin enhancer binding factor 3, 90kDa
	NM_004516	217805_at	1.30	8.24E-03	interleukin enhancer binding factor 3, 90kDa
INDO	M34455	210029_at	1.72	4.07E-03	indoleamine-pyrrole 2,3 dioxygenase
ISG15	NM_005101	205483_s_at	2.08	9.31E-03	ISG15 ubiquitin-like modifier
ISG20	NM_002201	204698_at	1.75	1.25E-03	interferon stimulated exonuclease gene 20kDa
	U88964	33304_at	1.72	1.46E-03	interferon stimulated exonuclease gene 20kDa
ITM2C	NM_030926	221004_s_at	1.43	2.64E-03	integral membrane protein 2C
ITPR1	NM_002222	203710_at	0.59	3.75E-03	inositol 1,4,5-triphosphate receptor, type 1
ITPR3	NM_002224	201189_s_at	1.31	7.39E-03	inositol 1,4,5-triphosphate receptor, type 3
KIF11	NM_004523	204444_at	1.58	8.12E-03	kinesin family member 11
KIF20A	NM_005733	218755_at	1.48	6.55E-03	kinesin family member 20A

KIF4A	NM_012310	218355_at	1.74	1.50E-03	kinesin family member 4A
KLHL2	NM_007246	219157_at	0.69	3.00E-03	kelch-like 2, Mayven (Drosophila)
KNTC2	NM_006101	204162_at	1.58	5.51E-03	kinetochore associated 2
KRT16	AF061812	209800_at	2.04	7.48E-03	keratin 16 (focal non-epidermolytic palmoplantar keratoderma)
KRT7	BC002700	209016_s_at	3.10	1.63E-03	keratin 7
KRTHB1	NM_002281	213711_at	2.23	4.76E-04	keratin, hair, basic, 1
LANCL2	NM_018697	218219_s_at	1.50	2.41E-03	LanC lantibiotic synthetase component C-like 2 (bacterial)
LAPTM4B	AW149681	208767_s_at	1.72	1.29E-04	lysosomal associated protein transmembrane 4 beta
	NM_018407	208029_s_at	1.81	1.07E-03	lysosomal associated protein transmembrane 4 beta
	T15777	214039_s_at	1.61	3.51E-03	lysosomal associated protein transmembrane 4 beta
LEPR	U50748	209894_at	0.67	9.54E-03	leptin receptor
LMNB1	NM_005573	203276_at	1.59	1.14E-03	lamin B1
LONPL	AI971258	221833_at	0.58	2.47E-03	peroxisomal LON protease like
LRPAP1	NM_002337	201186_at	0.75	7.36E-03	low density lipoprotein receptor-related protein associated protein 1
MAD2L1	NM_002358	203362_s_at	1.71	6.33E-03	MAD2 mitotic arrest deficient-like 1 (yeast)
MAF	NM_005360	206363_at	0.70	4.09E-03	v-maf musculoaponeurotic fibrosarcoma oncogene homolog (avian)
MAN1C1	NM_020379	218918_at	0.70	1.50E-03	mannosidase, alpha, class 1C, member 1
MAN2B2	AW954107	214703_s_at	0.64	6.51E-04	mannosidase, alpha, class 2B, member 2
MANBA	NM_005908	203778_at	0.76	8.57E-03	mannosidase, beta A, lysosomal
MAOA	AA923354	212741_at	0.67	1.66E-03	monoamine oxidase A
	NM_000240	204388_s_at	0.73	9.09E-03	monoamine oxidase A
MAP3K12	BE222201	205447_s_at	0.79	7.57E-03	mitogen-activated protein kinase kinase kinase 12
MAP3K15	BF739979	200979_at	1.39	9.61E-04	mitogen-activated protein kinase kinase kinase 15
MARCKSL1	NM_023009	200644_at	1.50	3.62E-03	MARCKS-like 1
MAST4	AB002301	40016_g_at	0.65	7.35E-03	microtubule associated serine/threonine kinase family member 4
MBNL2	NM_005757	205018_s_at	1.31	7.78E-03	muscleblind-like 2 (Drosophila)
MCM2	NM_004526	202107_s_at	1.58	3.87E-03	MCM2 minichromosome maintenance deficient 2, mitotin
MCM4	AI859865	222037_at	1.62	1.48E-03	MCM4 minichromosome maintenance deficient 4
	AI859865	222036_s_at	1.51	4.53E-03	MCM4 minichromosome maintenance deficient 4
ME3	NM_006680	204663_at	0.62	1.14E-04	malic enzyme 3, NADP(+)-dependent, mitochondrial
MELK	NM_014791	204825_at	2.05	1.75E-04	maternal embryonic leucine zipper kinase
MEOX2	NM_005924	206201_s_at	0.66	7.71E-03	mesenchyme homeobox 2
MFSD7	AK025922	214269_at	0.73	4.85E-03	major facilitator superfamily domain containing 7
MKL2	NM_014048	218259_at	0.76	8.99E-03	MKL/myocardin-like 2
MLH1	NM_000249	202520_s_at	0.79	9.03E-03	mutL homolog 1, colon cancer, nonpolyposis type 2 (E. coli)
MLLT3	NM_004529	204918_s_at	1.57	4.08E-03	myeloid/lymphoid or mixed-lineage leukemia (trithorax homolog, Drosophila); translocated to, 3
MOAP1	AK024029	212508_at	0.70	2.49E-03	modulator of apoptosis 1
MORC2	AC004542	216863_s_at	1.30	9.85E-03	MORC family CW-type zinc finger 2

MPPE1	BC002877	209858_x_at	0.83	9.69E-03	metallophosphoesterase 1
MR1	NM_001531	207565_s_at	0.71	5.58E-04	major histocompatibility complex, class I-related
	AF010446	210223_s_at	0.81	2.32E-03	major histocompatibility complex, class I-related
	AF031469	210224_at	0.82	9.56E-03	major histocompatibility complex, class I-related
MRPL12	NM_002949	203931_s_at	1.47	2.61E-04	mitochondrial ribosomal protein L12
MRPL19	NM_014763	203465_at	1.36	6.55E-03	mitochondrial ribosomal protein L19
MRPL2	NM_015950	218887_at	1.31	2.78E-03	mitochondrial ribosomal protein L2
MRPL42	BE782148	217919_s_at	1.32	5.49E-03	mitochondrial ribosomal protein L42
MRPS12	NM_021107	204331_s_at	1.70	2.34E-04	mitochondrial ribosomal protein S12
MT1X	NM_005952	208581_x_at	1.36	7.38E-03	metallothionein 1X
MT2A	NM_005953	212185_x_at	1.36	3.56E-03	metallothionein 2A
MTAP	NM_002451	204956_at	1.34	3.94E-03	methylthioadenosine phosphorylase
MTCP1	Z24459	216862_s_at	0.76	3.70E-03	mature T-cell proliferation 1
MTFR1	BF214329	203207_s_at	1.56	4.89E-03	mitochondrial fission regulator 1
	NM_014637	203208_s_at	1.37	9.95E-03	mitochondrial fission regulator 1
MTHFD2	NM_006636	201761_at	1.48	9.59E-04	methylenetetrahydrofolate dehydrogenase 2
MULK	NM_018238	218568_at	1.33	8.10E-04	multiple substrate lipid kinase
MYBL1	AW592266	213906_at	1.92	9.40E-03	v-myb myeloblastosis viral oncogene homolog (avian)-like 1
MYNN	NM_018657	218926_at	1.30	8.33E-03	myoneurin
MYO10	NM_012334	201976_s_at	1.47	3.98E-03	myosin X
NAT10	NM_024662	217884_at	1.31	4.17E-03	N-acetyltransferase 10
NBN	NM_002485	202907_s_at	1.64	4.51E-03	nibrin
NEK3	AI191920	213116_at	0.75	8.44E-03	NIMA (never in mitosis gene a)-related kinase 3
NENF	NM_013349	218407_x_at	0.68	4.31E-04	neuron derived neurotrophic factor
NLGN4X	AI338338	221933_at	0.55	4.15E-04	neuroligin 4, X-linked
NME1	NM_000269	201577_at	1.69	1.11E-03	non-metastatic cells 1, protein (NM23A) expressed in
NOL6	NM_022917	218199_s_at	1.21	7.46E-03	nucleolar protein family 6 (RNA-associated)
NPM3	NM_006993	205129_at	1.35	3.00E-03	nucleophosmin/nucleoplasmin, 3
NR4A2	S77154	216248_s_at	0.48	6.71E-04	nuclear receptor subfamily 4, group A, member 2
	AI935096	204621_s_at	0.58	1.34E-03	nuclear receptor subfamily 4, group A, member 2
	NM_006186	204622_x_at	0.52	1.42E-03	nuclear receptor subfamily 4, group A, member 2
NRIP3	NM_020645	219557_s_at	0.51	4.92E-04	nuclear receptor interacting protein 3
NUP205	AW008531	212247_at	1.45	1.06E-03	nucleoporin 205kDa
OAS1	NM_016816	202869_at	1.86	9.39E-04	2',5'-oligoadenylate synthetase 1, 40/46kDa
OAS2	NM_016817	204972_at	1.56	9.50E-04	2'-5'-oligoadenylate synthetase 2, 69/71kDa
ODC1	NM_002539	200790_at	1.81	6.70E-04	ornithine decarboxylase 1
OLFM1	NM_006334	205591_at	0.76	4.12E-03	olfactomedin 1
	R38389	213131_at	0.67	7.06E-03	olfactomedin 1
OR7E37P	AW874308	217499_x_at	1.50	2.48E-03	olfactory receptor, family 7, subfamily E, member 37
OSBPL8	AL049923	212582_at	0.64	5.61E-04	oxysterol binding protein-like 8
PADI2	AL049569	209791_at	1.50	6.51E-03	peptidyl arginine deiminase, type II

PAICS	NM_006452	201014_s_at	1.54	9.32E-03	phosphoribosylaminoimidazole succinocarboxamide synthetase
PARP12	NM_022750	218543_s_at	1.54	2.70E-03	poly (ADP-ribose) polymerase family, member 12
PCID1	NM_006360	202232_s_at	1.40	3.21E-03	PCI domain containing 1 (herpesvirus entry mediator)
PDE4A	NM_006202	204735_at	0.65	2.70E-03	phosphodiesterase 4A, cAMP-specific
PDIA5	NM_006810	203857_s_at	1.37	8.36E-03	protein disulfide isomerase family A, member 5
PDIA6	BE910010	208638_at	1.39	1.50E-03	protein disulfide isomerase family A, member 6
	BC001312	208639_x_at	1.45	3.51E-03	protein disulfide isomerase family A, member 6
	AK026926	216640_s_at	1.46	5.00E-03	protein disulfide isomerase family A, member 6
	NM_005742	207668_x_at	1.37	7.95E-03	protein disulfide isomerase family A, member 6
PEX6	D83703	320_at	1.32	7.11E-03	peroxisomal biogenesis factor 6
PFAAP5	AW084068	214753_at	0.73	3.61E-03	phosphonoformate immuno-associated protein 5
PHF16	NM_014735	204866_at	1.74	2.06E-03	PHD finger protein 16
PHGDH	NM_006623	201397_at	1.60	4.59E-03	phosphoglycerate dehydrogenase
PLAA	AF145020	209533_s_at	1.44	2.92E-04	phospholipase A2-activating protein
PLAGL1	NM_002656	207002_s_at	0.58	3.50E-03	pleiomorphic adenoma gene-like 1 (Zac1)
PLCH1	AW665865	214745_at	1.29	7.64E-03	phospholipase C, eta 1
POU2AF1	NM_006235	205267_at	2.31	2.88E-03	POU domain, class 2, associating factor 1
PPA1	NM_021129	217848_s_at	1.39	4.73E-03	pyrophosphatase (inorganic) 1
PPARD	L07592	37152_at	1.23	9.11E-03	peroxisome proliferative activated receptor, delta
PPAT	U00238	209434_s_at	1.42	1.13E-03	phosphoribosyl pyrophosphate amidotransferase
PPIG	AW340788	208993_s_at	0.73	2.37E-03	peptidylprolyl isomerase G (cyclophilin G)
PPM1G	NM_002707	200913_at	1.26	3.53E-03	protein phosphatase 1G (formerly 2C), magnesium-dependent, gamma isoform
PRC1	NM_003981	218009_s_at	1.78	2.68E-03	protein regulator of cytokinesis 1
PRO1843	NM_018507	219599_at	0.61	9.27E-05	hypothetical protein PRO1843
PRPF4B	Z25435	211090_s_at	1.23	7.74E-03	PRP4 pre-mRNA processing factor 4 homolog B (yeast)
PSMB9	NM_002800	204279_at	1.77	3.04E-03	proteasome (prosome, macropain) subunit, beta type, 9 (large multifunctional peptidase 2)
PSMC2	NM_002803	201068_s_at	1.30	8.81E-03	proteasome (prosome, macropain) 26S subunit, ATPase, 2
PSMD12	AI446530	202352_s_at	1.48	3.17E-03	proteasome (prosome, macropain) 26S subunit, non-ATPase, 12
	NM_002816	202353_s_at	1.35	7.71E-03	proteasome (prosome, macropain) 26S subunit, non-ATPase, 12
PSMD3	NM_002809	201388_at	1.65	8.71E-03	proteasome (prosome, macropain) 26S subunit, non-ATPase, 3
PSPHL	NM_003832	205048_s_at	5.95	3.46E-10	phosphoserine phosphatase-like
PTP4A2	BF795101	208615_s_at	0.71	7.78E-03	protein tyrosine phosphatase type IVA, member 2
PTPRK	NM_002844	203038_at	1.80	6.30E-03	protein tyrosine phosphatase, receptor type, K
PTPRN2	AF007555	203030_s_at	0.77	6.79E-03	protein tyrosine phosphatase, receptor type, N polypeptide 2
PTS	M97655	209694_at	1.38	8.04E-03	6-pyruvoyltetrahydropterin synthase

PUS7	NM_019042	218984_at	1.54	2.21E-04	pseudouridylate synthase 7 homolog
QDPR	BC000576	209123_at	0.57	2.74E-03	quinoid dihydropteridine reductase
RAB13	NM_002870	202252_at	0.75	9.63E-04	RAB13, member RAS oncogene family
RAD51AP1	BE966146	204146_at	1.54	6.27E-03	RAD51 associated protein 1
RAPGEF3	U78168	210051_at	0.76	1.99E-03	Rap guanine nucleotide exchange factor (GEF) 3
RB1	NM_000321	203132_at	0.73	4.39E-03	retinoblastoma 1 (including osteosarcoma)
RBM14	NM_006328	204178_s_at	1.26	6.18E-03	RNA binding motif protein 14
RCOR3	NM_018254	218344_s_at	0.73	9.51E-03	REST corepressor 3
REPS2	NM_004726	205645_at	0.62	4.44E-04	RALBP1 associated Eps domain containing 2
RIS1	BF062629	213338_at	1.71	4.33E-03	Ras-induced senescence 1
RNASE4	AI761728	213397_x_at	0.52	1.47E-03	ribonuclease, RNase A family, 4
	NM_002937	205158_at	0.68	9.49E-03	ribonuclease, RNase A family, 4
ROD1	AW190873	214697_s_at	1.38	3.48E-03	ROD1 regulator of differentiation 1
RP6-213H19.1	NM_016542	218499_at	1.65	5.62E-03	Mst3 and SOK1-related kinase
RPIA	AI692341	212973_at	1.34	5.73E-03	ribose 5-phosphate isomerase A (ribose 5-phosphate epimerase)
RPL13	AI186735	212734_x_at	0.77	7.83E-03	ribosomal protein L13
RPP40	NM_006638	213427_at	1.49	1.84E-03	ribonuclease P 40kDa subunit
RPS19	BE259729	213414_s_at	1.27	3.21E-03	ribosomal protein S19
	NM_001022	202649_x_at	1.23	8.90E-03	ribosomal protein S19
RRAS2	AI431643	212590_at	1.38	2.24E-03	related RAS viral (r-ras) oncogene homolog 2
RRM2	BE966236	201890_at	2.43	2.12E-04	ribonucleotide reductase M2 polypeptide
	BC001886	209773_s_at	2.02	5.79E-04	ribonucleotide reductase M2 polypeptide
RSAD2	AI337069	213797_at	2.14	9.29E-04	radical S-adenosyl methionine domain containing 2
RTCD1	NM_003729	203594_at	1.41	6.57E-03	RNA terminal phosphate cyclase domain 1
S100P	NM_005980	204351_at	3.15	1.11E-03	S100 calcium binding protein P
SACM1L	NM_014016	202797_at	0.71	7.31E-04	SAC1 suppressor of actin mutations 1-like
SAT	BE971383	213988_s_at	1.39	7.96E-03	spermidine/spermine N1-acetyltransferase
SCGB2A1	NM_002407	205979_at	0.30	7.84E-04	secretoglobin, family 2A, member 1
SCP2	AI753792	212589_at	1.47	9.50E-03	sterol carrier protein 2
SCUBE2	AI424243	219197_s_at	0.44	1.75E-03	signal peptide, CUB domain, EGF-like 2
SDC1	NM_002997	201287_s_at	1.56	8.16E-03	syndecan 1
SERBP1	NM_015640	217725_x_at	1.32	8.93E-03	SERPINE1 mRNA binding protein 1
SEZ6L2	NM_012410	218720_x_at	0.70	8.69E-03	seizure related 6 homolog (mouse)-like 2
SF3B1	AW003030	214305_s_at	0.77	5.44E-03	splicing factor 3b, subunit 1, 155kDa
SF3B3	NM_012426	200687_s_at	1.39	4.52E-03	splicing factor 3b, subunit 3, 130kDa
SFRS6	AL031681	208804_s_at	0.76	7.98E-03	splicing factor, arginine/serine-rich 6
SH3BGRL	AL515318	201311_s_at	0.68	5.79E-03	SH3 domain binding glutamic acid-rich protein like
	NM_003022	201312_s_at	0.71	9.85E-03	SH3 domain binding glutamic acid-rich protein like
SHMT2	AW190316	214095_at	1.31	1.33E-03	serine hydroxymethyltransferase 2 (mitochondrial)
	NM_005412	214437_s_at	1.46	3.18E-03	serine hydroxymethyltransferase 2 (mitochondrial)
SLC27A3	BC003654	222217_s_at	0.71	2.29E-04	solute carrier family 27 (fatty acid transporter), member 3
SLC30A1	AI972416	212907_at	0.73	8.31E-03	solute carrier family 30 (zinc transporter), member 1
SLC35F2	NM_017515	218826_at	1.33	7.74E-04	solute carrier family 35, member F2

SLC39A7	NM_006979	202667_s_at	1.60	1.20E-03	solute carrier family 39 (zinc transporter), member 7
SLC5A6	NM_021095	204087_s_at	1.58	1.85E-04	solute carrier family 5 (sodium-dependent vitamin transporter), member 6
SLC7A1	AA148507	212295_s_at	1.40	7.71E-04	solute carrier family 7 (cationic amino acid transporter, y+ system), member 1
SLC7A5	AB018009	201195_s_at	2.05	1.14E-03	solute carrier family 7 (cationic amino acid transporter, y+ system), member 5
SMARCA4	AF254822	215714_s_at	1.33	3.95E-03	SWI/SNF related, matrix associated, actin dependent regulator of chromatin, subfamily a, member 4
SMARCD3	NM_003078	204099_at	0.63	1.87E-03	SWI/SNF related, matrix associated, actin dependent regulator of chromatin, subfamily d, member 3
SNED1	N73970	213488_at	0.76	7.42E-03	sushi, nidogen and EGF-like domains 1
SNX1	NM_003099	201716_at	0.78	4.79E-03	sorting nexin 1
SOS1	L13857	212777_at	1.52	5.21E-05	son of sevenless homolog 1 (Drosophila)
SOX11	AI360875	204913_s_at	2.41	8.37E-04	SRY (sex determining region Y)-box 11
	AW157202	204914_s_at	1.83	2.09E-03	SRY (sex determining region Y)-box 11
SOX4	AL136179	201417_at	1.50	4.55E-03	SRY (sex determining region Y)-box 4
SPAG5	NM_006461	203145_at	1.55	8.04E-04	sperm associated antigen 5
SPATA20	NM_022827	218164_at	0.70	4.13E-03	spermatogenesis associated 20
SPCS3	NM_021928	218817_at	1.27	4.13E-03	signal peptidase complex subunit 3 homolog (S. cerevisiae)
SPP1	M83248	209875_s_at	0.40	2.46E-03	secreted phosphoprotein 1 (osteopontin, bone sialoprotein I, early T-lymphocyte activation 1)
SRPK1	NM_003137	202200_s_at	1.37	6.00E-03	SFRS protein kinase 1
SRPR	NM_003139	200918_s_at	1.27	7.94E-03	signal recognition particle receptor ('docking protein')
SSBP1	NM_003143	202591_s_at	1.46	2.39E-03	single-stranded DNA binding protein 1
SSR1	AW006345	200890_s_at	1.42	5.57E-03	signal sequence receptor, alpha (translocon-associated protein alpha)
STAG2	BC001765	209023_s_at	0.76	6.96E-03	stromal antigen 2
	BC000795	221610_s_at	1.49	3.75E-03	signal-transducing adaptor protein-2
STAT1	NM_007315	200887_s_at	1.69	6.52E-04	signal transducer and activator of transcription 1, 91kDa
	BC002704	209969_s_at	1.84	3.24E-03	signal transducer and activator of transcription 1, 91kDa
STOM	M81635	201061_s_at	0.68	6.67E-03	stomatin
STYK1	NM_018423	220030_at	1.79	1.00E-03	serine/threonine/tyrosine kinase 1
SYCP2	NM_014258	206546_at	0.64	2.56E-03	synaptonemal complex protein 2
SYT17	NM_016524	205613_at	0.57	5.42E-04	synaptotagmin XVII
TACSTD1	NM_002354	201839_s_at	2.50	2.44E-03	tumor-associated calcium signal transducer 1
TAP1	NM_000593	202307_s_at	1.78	5.89E-04	transporter 1, ATP-binding cassette, sub-family B (MDR/TAP)
TAP2	M74447	204769_s_at	1.48	2.15E-03	transporter 2, ATP-binding cassette, sub-family B (MDR/TAP)
	NM_000544	204770_at	1.22	5.66E-03	transporter 2, ATP-binding cassette, sub-family B (MDR/TAP)
TBC1D5	AI300084	201814_at	0.72	1.64E-04	TBC1 domain family, member 5

TBC1D8	NM_007063	204526_s_at	0.72	8.90E-03	TBC1 domain family, member 8 (with GRAM domain)
TBRG4	NM_004749	220789_s_at	1.27	4.37E-03	transforming growth factor beta regulator 4
TCP1	BF224073	222010_at	1.43	7.37E-04	t-complex 1
TDRD3	AU156998	214028_x_at	0.77	6.90E-03	tudor domain containing 3
	NM_030794	208089_s_at	0.71	9.97E-03	tudor domain containing 3
TES	NM_015641	202720_at	1.43	4.15E-03	testis derived transcript (3 LIM domains)
TFDP1	R60866	212330_at	1.46	3.75E-03	transcription factor Dp-1
THBD	NM_000361	203887_s_at	0.49	1.20E-05	thrombomodulin
THRAP4	AI023317	213043_s_at	1.72	4.54E-04	thyroid hormone receptor associated protein 4
TIMM23	NM_006327	218118_s_at	1.33	5.20E-03	translocase of inner mitochondrial membrane 23 homolog
TM2D1	AA012917	213883_s_at	0.78	8.34E-03	TM2 domain containing 1
TMED2	NM_006815	204427_s_at	1.34	4.53E-03	transmembrane emp24 domain trafficking protein 2
TMEM121	NM_025268	219663_s_at	0.80	8.65E-03	transmembrane protein 121
TMEM127	NM_017849	219460_s_at	0.81	3.75E-03	transmembrane protein 127
TMEM132A	NM_017870	218834_s_at	1.44	2.97E-03	transmembrane protein 132A
TMPO	AW272611	203432_at	1.48	3.58E-04	thymopoietin
TNXB	BE044614	213451_x_at	0.63	3.01E-03	tenascin XB
	M25813	216333_x_at	0.67	4.73E-03	tenascin XB
TOP2A	AU159942	201291_s_at	1.89	8.91E-03	topoisomerase (DNA) II alpha 170kDa
TOPORS	NM_005802	204071_s_at	1.29	9.69E-03	topoisomerase I binding, arginine/serine-rich
TOR1AIP1	AK023204	212408_at	0.78	5.87E-03	torsin A interacting protein 1
	AK021613	212409_s_at	0.83	7.87E-03	torsin A interacting protein 1
TRAF5	NM_004619	204352_at	0.65	4.36E-03	TNF receptor-associated factor 5
TRAK1	NM_014965	202080_s_at	0.77	6.71E-03	trafficking protein, kinesin binding 1
TRIAP1	NM_016399	218403_at	1.37	5.01E-03	TP53 regulated inhibitor of apoptosis 1
TRPC1	NM_003304	205803_s_at	0.73	8.58E-03	transient receptor potential cation channel, subfamily C, member 1
TRPV6	NM_014274	206827_s_at	1.49	2.00E-03	transient receptor potential cation channel, subfamily V, member 6
TSPAN31	NM_005981	203227_s_at	0.72	1.78E-03	tetraspanin 31
TSPAN4	BC000389	209263_x_at	0.73	5.08E-03	tetraspanin 4
TSPAN8	NM_004616	203824_at	2.09	7.36E-03	tetraspanin 8
TSPYL5	AI096375	213122_at	1.67	3.97E-03	TSPY-like 5
TSR1	NM_018128	218156_s_at	1.37	4.55E-03	TSR1, 20S rRNA accumulation, homolog (S. cerevisiae)
TTK	NM_003318	204822_at	1.62	2.38E-03	TTK protein kinase
U2AF2	NM_007279	218381_s_at	1.32	5.88E-03	U2 small nuclear RNA auxiliary factor 2
UBAP2	NM_018449	219192_at	1.61	1.31E-04	ubiquitin associated protein 2
	AK026088	221839_s_at	1.34	1.98E-03	ubiquitin associated protein 2
UBE2C	NM_007019	202954_at	1.44	2.61E-03	ubiquitin-conjugating enzyme E2C
UBE2I	AL031714	208760_at	0.72	2.11E-03	Ubiquitin-conjugating enzyme E2I (UBC9 homolog, yeast)
UBE2N	BG290646	212751_at	1.39	1.48E-03	ubiquitin-conjugating enzyme E2N (UBC13 homolog, yeast)
UROD	M14016	208970_s_at	0.78	9.59E-03	uroporphyrinogen decarboxylase
USP4	AF017306	211800_s_at	0.80	7.51E-03	ubiquitin specific peptidase 4 (proto-oncogene)
VCP	AF100752	208649_s_at	1.25	7.74E-03	valosin-containing protein

VEGF	AF022375	210512_s_at	1.59	1.31E-03	vascular endothelial growth factor
VPS37B	BC005882	221704_s_at	1.39	1.63E-03	vacuolar protein sorting 37 homolog B
WDR67	AI017564	214061_at	1.30	5.02E-03	WD repeat domain 67
WSB2	NM_018639	201760_s_at	1.34	8.03E-03	WD repeat and SOCS box-containing 2
XK	NM_021083	206698_at	1.53	9.29E-04	X-linked Kx blood group (McLeod syndrome)
XPOT	AI984005	212160_at	1.42	1.38E-03	exportin, tRNA (nuclear export receptor for tRNAs)
YWHAQ	NM_006826	200693_at	1.51	1.73E-04	tyrosine 3-monooxygenase
YY1	Z14077	201901_s_at	0.81	9.50E-03	YY1 transcription factor
ZBED4	NM_014838	204799_at	1.37	1.56E-03	zinc finger, BED-type containing 4
ZBTB38	NM_024724	219221_at	0.72	1.23E-03	zinc finger and BTB domain containing 38
ZBTB5	NM_014872	203026_at	1.35	2.09E-03	zinc finger and BTB domain containing 5
ZC3HAV1	NM_020119	220104_at	1.29	4.36E-03	zinc finger CCCH-type, antiviral 1
ZHX3	AB007855	217367_s_at	0.81	4.82E-03	zinc fingers and homeoboxes 3
ZIC1	NM_003412	206373_at	1.85	8.91E-04	Zic family member 1
ZMYND11	BE250417	202136_at	0.76	3.95E-03	zinc finger, MYND domain containing 11
ZNF277	NM_021994	218645_at	1.35	1.99E-03	zinc finger protein 277
ZNF281	NM_012482	218401_s_at	0.62	9.92E-04	zinc finger protein 281
ZNF302	NM_018443	218490_s_at	0.67	5.20E-03	zinc finger protein 302
ZNF331	NM_018555	219228_at	0.73	2.18E-03	zinc finger protein 331
ZNF395	NM_018660	221123_x_at	0.69	3.46E-03	zinc finger protein 395
ZNF688	AI095896	213527_s_at	0.76	3.41E-03	zinc finger protein 688

African-Americans (n = 18) versus European-Americans (n = 17; reference)
