# Supplementary material for: Differences in the Tumor Microenvironment between African-American and European-American Breast Cancer Patients
Source: PLoS One. 2009 Feb 19;4(2):e4531. doi: 10.1371/journal.pone.0004531 (PMC2638012; doi:10.1371/journal.pone.0004531)
Supplement: Table S2 — (0.03 MB PDF) [file pone.0004531.s003.pdf]

Table S2. Top genes differently expressed by race/ethnicity in ER-positive tumors*

Gene Name	GenBank ID	Affy ID	Fold change	*P* value	Gene Title
IGL	X57812	214677_x_at	14.3	0.0003	immunoglobulin lambda locus
PDZK1	NM_002614	205380_at	12.51	0.001	PDZ domain containing 1
IGH	M87789	211430_s_at	8.47	0.0005	immunoglobulin heavy locus
CXCL10	NM_001565	204533_at	6.36	0.0001	chemokine (C-X-C motif) ligand 10
IGJ	AV733266	212592_at	6.27	0.001	Immunoglobulin J polypeptide
CXCL11	AF030514	210163_at	5.86	0.0005	chemokine (C-X-C motif) ligand 11
IFIT1	NM_001548	203153_at	5.55	0.004	interferon-induced protein with tetratricopeptide repeats 1
DIO1	NM_000792	206457_s_at	5.55	0.001	deiodinase, iodothyronine, type I
ISG15	NM_005101	205483_s_at	4.98	0.008	ISG15 ubiquitin-like modifier
RSAD2	AI337069	213797_at	4.79	0.002	radical S-adenosyl methionine domain containing 2
IGKC	L14458	211644_x_at	4.78	0.00007	immunoglobulin kappa constant
IFI27	NM_005532	202411_at	3.93	0.003	interferon, alpha-inducible protein 27
OAS1	NM_002534	205552_s_at	3.8	0.003	2',5'-oligoadenylate synthetase 1, 40/46kDa
IGKV1D	AW408194	216207_x_at	3.75	0.0002	immunoglobulin kappa variable 1D-13
OASL	NM_003733	205660_at	3.69	0.001	2'-5'-oligoadenylate synthetase-like
LEF1	AF288571	221558_s_at	3.66	0.004	lymphoid enhancer-binding factor 1
SERPINA6	NM_001756	206325_at	3.62	0.002	serpin peptidase inhibitor, clade A, member 6
IFIH1	NM_022168	219209_at	3.51	0.001	interferon induced with helicase C domain 1
TNFSF10	U57059	202687_s_at	3.41	0.002	tumor necrosis factor (ligand) superfamily, member 10
RTP4	NM_022147	219684_at	3.4	0.003	receptor transporter protein 4
SERPINI1	NM_005025	205352_at	3.31	0.009	serpin peptidase inhibitor, clade I (neuroserpin), member 1
STAT1	BC002704	209969_s_at	3.31	0.004	signal transducer and activator of transcription 1, 91kDa
OAS3	NM_006187	218400_at	3.3	0.003	2'-5'-oligoadenylate synthetase 3, 100kDa
TUBA3	AF141347	209118_s_at	0.49	0.001	tubulin, alpha 3
HLA-DPA1	AI128225	213537_at	0.49	0.01	major histocompatibility complex, class II, DP alpha 1
CD59	BE379006	212463_at	0.49	0.0005	CD59 molecule, complement regulatory protein
KCNMA1	U11058	221584_s_at	0.48	0.003	potassium large conductance calcium-activated channel, subfamily M, alpha member 1
STOM	M81635	201061_s_at	0.47	0.008	stomatin
PHACTR2	AA551142	204048_s_at	0.47	0.003	phosphatase and actin regulator 2
EFHC1	NM_018100	219833_s_at	0.47	0.007	EF-hand domain (C-terminal) containing 1
LEPR	U50748	209894_at	0.46	0.008	leptin receptor
THBD	NM_000361	203887_s_at	0.45	0.003	thrombomodulin
CDKN1C	N33167	213348_at	0.44	0.004	Cyclin-dependent kinase inhibitor 1C (p57, Kip2)
GALNAC4S	NM_014863	203066_at	0.44	0.006	B cell RAG associated protein
GSN	NM_000177	200696_s_at	0.42	0.01	gelsolin
FZD7	NM_003507	203706_s_at	0.4	0.004	frizzled homolog 7
RNASE4	AI761728	213397_x_at	0.35807654	0.009	ribonuclease, RNase A family, 4

NR4A2	S77154	216248_s_at	0.354337112	0.007	nuclear receptor subfamily 4, group A, member 2
FHL1	NM_001449	201540_at	0.353196078	0.006	four and a half LIM domains 1
COLEC12	NM_030781	221019_s_at	0.343123413	0.006	collectin sub-family member 12
SLCO3A1	NM_013272	219229_at	0.338252707	0.003	solute carrier organic anion transporter family, member 3A1
EFEMP1	NM_004105	201843_s_at	0.331626982	0.008	EGF-containing fibulin-like extracellular matrix protein 1
CDC42BPA	NM_003607	214464_at	0.271887274	0.00006	CDC42 binding protein kinase alpha (DMPK-like)
PEG3	AL042588	209242_at	0.204398976	0.0001	paternally expressed 3
AGTR1	NM_000685	205357_s_at	0.201216164	0.005	angiotensin II receptor, type 1
KRT15	NM_002275	204734_at	0.174272463	0.006	keratin 15

* Gene list for microdissected tumor epithelium comparing African-American and European-American patients (= reference). Genes that were found to be differently expressed ($P \leq 0.01$) in the tumor epithelium of ER-negative tumors are excluded.
