# Supplementary material for: Differences in the Tumor Microenvironment between African-American and European-American Breast Cancer Patients
Source: PLoS One. 2009 Feb 19;4(2):e4531. doi: 10.1371/journal.pone.0004531 (PMC2638012; doi:10.1371/journal.pone.0004531)
Supplement: Table S3 — (0.02 MB PDF) [file pone.0004531.s004.pdf]

Table S3. Top genes differently expressed by race/ethnicity in ER-negative tumors*

Gene Name	GenBank ID	Affy ID	Fold change	*P* value	Gene Title
HLA-DQA1	X00452	213831_at	4.0	0.0002	major histocompatibility complex, class II, DQ alpha 1
HLA-DQB1	M16276	209480_at	3.27	0.0002	major histocompatibility complex, class II, DQ beta 1
MMP1	NM_002421	204475_at	2.96	0.006	matrix metallopeptidase 1
INHBA	M13436	210511_s_at	2.87	0.0002	inhibin, beta A
FABP7	NM_001446	205029_s_at	2.71	0.004	fatty acid binding protein 7, brain
C3orf52	NM_024616	219474_at	2.65	0.0001	chromosome 3 open reading frame 52
COL10A1	X98568	217428_s_at	2.56	0.002	collagen, type X, alpha 1
DSP	NM_004415	200606_at	2.51	0.0003	desmoplakin
TPD52	BE974098	201689_s_at	2.24	0.003	tumor protein D52
VDAC1	AL515918	212038_s_at	2.12	0.0002	voltage-dependent anion channel 1
CMAS	NM_018686	218111_s_at	2.06	0.002	cytidine monophosphate N-acetylneuraminic acid synthetase
RP6	NM_016542	218499_at	2.04	0.001	Mst3 and SOK1-related kinase
HSP90AB1	AI218219	214359_s_at	2.03	0.001	heat shock protein 90kDa alpha (cytosolic), class B member 1
RRM2	BE966236	201890_at	2.02	0.002	ribonucleotide reductase M2 polypeptide
MYO6	NM_004999	203216_s_at	2.0	0.0001	myosin VI
PARVB	AA187563	37966_at	0.62	0.0006	parvin, beta
SLC26A2	AI025519	205097_at	0.62	0.006	solute carrier family 26 (sulfate transporter), member 2
BTG1	AL535380	200920_s_at	0.61	0.003	B-cell translocation gene 1, anti-proliferative
MCAM	AF089868	209087_x_at	0.61	0.006	melanoma cell adhesion molecule
PLTP	NM_006227	202075_s_at	0.61	0.0007	phospholipid transfer protein
MS4A6A	NM_022349	219666_at	0.60	0.003	membrane-spanning 4-domains, subfamily A, member 6A
RORA	L14611	210479_s_at	0.55	0.002	RAR-related orphan receptor A
CCL14	NM_004166	205392_s_at	0.53	0.005	chemokine (C-C motif) ligand 14
NLGN4X	AI338338	221933_at	0.52	0.002	neuroligin 4, X-linked
MRC1	NM_002438	204438_at	0.51	0.001	mannose receptor, C type 1
SMARCD3	NM_003078	204099_at	0.5	0.002	SWI/SNF related, matrix associated, actin dependent regulator of chromatin, subfamily d, member 3
CD302	NM_014880	203799_at	0.5	0.002	CD302
SELENBP1	NM_003944	214433_s_at	0.49	0.002	selenium binding protein 1
MATN2	NM_002380	202350_s_at	0.39	0.003	matrilin 2
HLA-DRB4	BC005312	209728_at	0.32	0.001	major histocompatibility complex, class II, DR beta 4

* Gene list for microdissected tumor epithelium comparing African-American and European-American patients (= reference). Genes that were found to be differently expressed ($P \leq 0.01$) in the tumor epithelium of ER-positive tumors are excluded.
