# Supplementary material for: Differences in the Tumor Microenvironment between African-American and European-American Breast Cancer Patients
Source: PLoS One. 2009 Feb 19;4(2):e4531. doi: 10.1371/journal.pone.0004531 (PMC2638012; doi:10.1371/journal.pone.0004531)
Supplement: Table S4 — (0.01 MB PDF) [file pone.0004531.s005.pdf]

Table S4. Cel file ID numbers

#	Race	Chip	Stroma_array (GEO ID)	Tumor_array (GEO ID)	TNM Stage	ER Status
1	EA	HGU133A	GSM136339	GSM136386	I	POS
2	AA	HGU133A	GSM136340	GSM136387	IIA	NEG
3	AA	HGU133A	N/A	GSM136388	IIA	POS
4	EA	HGU133A	GSM136341	GSM136389	I	POS
5	EA	HGU133A	GSM136342	GSM136390	IIA	POS
6	AA	HGU133A	GSM136343	GSM136391	IIA	NEG
7	EA	HGU133A	GSM136344	GSM136392	IIB	POS
8	EA	HGU133A	GSM136345	GSM136393	IIA	POS
9	EA	HGU133A	GSM136347	GSM136395	IIA	POS
10	AA	HGU133A	GSM136348	GSM136396	IIA	POS
11	AA	HGU133A	GSM136351	GSM136399	IIB	NEG
12	EA	HGU133A	GSM136346	GSM136394	IIA	NEG
13	EA	HGU133A	GSM136349	GSM136397	IIB	POS
14	EA	HGU133A	GSM136350	GSM136398	IIIA	N/A
15	EA	HGU133A	GSM136352	GSM136400	IIB	NEG
16	AA	HGU133A	GSM136355	GSM136403	IIA	NEG
17	EA	HGU133A	GSM136356	GSM136404	I	POS
18	EA	HGU133A	GSM136357	GSM136405	IIB	NEG
19	AA	HGU133A	GSM136358	GSM136406	IIB	NEG
20	EA	HGU133A	GSM136359	GSM136407	IIB	POS
21	AA	HGU133A	GSM136360	GSM136408	IIB	NEG
22	EA	HGU133A	GSM136361	GSM136409	IIB	POS
23	EA	HGU133A	GSM136362	GSM136410	IIB	POS
24	AA	HGU133A	GSM136363	GSM136411	IIA	POS
25	EA	HGU133A	GSM136364	GSM136412	IIB	NEG
26	AA	HGU133A	GSM136365	GSM136413	I	NEG
27	AA	HGU133A	GSM136366	GSM136414	IIB	POS
28	AA	HGU133A	GSM136368	GSM136416	IIB	NEG
29	AA	HGU133A	GSM136369	GSM136417	IIA	NEG
30	AA	HGU133A	GSM136370	GSM136418	IIB	NEG
31	AA	HGU133A	GSM136372	GSM136420	IIA	NEG
32	AA	HGU133A	GSM136353	GSM136401	IIB	NEG
33	AA	HGU133A	GSM136354	GSM136402	IIA	POS
34	AA	HGU133A	GSM136367	GSM136415	IIIA	NEG
35	EA	HGU133A	GSM136371	GSM136419	IIB	NEG

AA = African American
EA = European-American
